# GraphLane: a spatio-temporal graph learning framework for lane localization

**DOI:** 10.3389/frai.2026.1946849

**Published:** 2026-10-21

**Authors:** V. Gunasekaran, J. Arun Pandian

**Affiliations:** 1 School of Computer Science Engineering and Information Systems, Vellore Institute of Technology, Vellore, India; 2 Centre for Artificial Intelligence Research, Vellore Institute of Technology, Vellore, India

**Keywords:** dynamic graph construction, geometry-aware optimization, graph learning, lane detection, lane localization, spatio-temporal learning

## Abstract

**Introduction:**

Lane localization is an important component of advanced driver assistance systems and autonomous driving, where accurate estimation of lane geometry is essential for safe vehicle navigation. Nevertheless, the current lane detection algorithms may face difficulties in complex driving situations due to poor visual feature extraction, unstable temporal prediction, and inadequate modeling of structural relations between lanes.

**Methods:**

In order to overcome these issues, this paper introduces the unified spatio-temporal graph learning approach that integrates spatial feature extraction, temporal context aggregation, uncertainty-driven graph formation, graph attention reasoning, and geometric optimization. The proposed GraphLane framework dynamically learns graph connections between lane keypoints while incorporating uncertainty estimation to suppress unreliable feature interactions and temporal consistency to improve structural stability across consecutive frames. A geometry-aware optimization strategy is further introduced to encourage smooth and physically consistent lane predictions. The framework was evaluated on the CULane test set using the proposed row-anchor-based lane-existence and localization evaluation.

**Results:**

Experimental results show that the proposed GraphLane framework achieves a lane-existence precision of 0.9769, recall of 0.8990, F1-score of 0.9363, and an average localization error of 19.28 pixels. Qualitative analysis shows that the framework produces smooth and consistent lane predictions under occlusions, shadows, dense traffic, and nighttime conditions.

**Discussion:**

Other analyses on learned graphs, computational complexity, and ablation studies also show that incorporating temporal context, adaptive graph reasoning, and geometrically informed optimizations is effective for lane localization.

## 1 Introduction

Lane detection has emerged as an essential element in advanced driver assistance systems (ADAS) and autonomous driving systems. Proper recognition of lanes is required for trajectory prediction and vehicle control. In recent times, deep convolutional neural network models have greatly helped in lane detection due to their ability to detect high-level features from raw images.

Given that lane markings consist of slender and elongated structures, standard object detection networks fail to extract such elements. Various networks have been introduced to solve this issue. Spatial dependencies in terms of rows and columns have been utilized to transfer information between the pixels to improve detection of long and continuous objects (Pan et al., 2018). Additionally, to satisfy the real-time requirements of autonomous driving, row-anchor-based classification networks and attention-based networks gained wide use (Qin et al., 2020; Tabelini et al., 2021). These anchor-based systems managed to find an equilibrium between computational efficiency and spatial accuracy through the transformation of lane detection into a row-wise selection task. Although significant advances have been made in this field, there are still problems associated with single-frame detection techniques, such as heavy occlusion due to traffic, absence of lane markings, and poor illumination. Spatio-temporal methods have been introduced to solve this problem by using past video frames, which will allow missing spatial information to be recovered from prior temporal information (Zhang et al., 2021). However, mere aggregation of temporal information may not be enough in the case of extreme visual degradation. In this case, the topology of the lane structure predicted may be quite unstable, causing erratic graph jitter and disconnection between the lanes across different frames. Although existing spatio-temporal and graph-based methods yielded promising results, there still exist three main challenges in lane detection that have not been addressed yet. First, in almost all graph-based methods, all visual features obtained from an image are considered to be equally trustworthy. In cases where images are affected by shadow, light, or occlusion caused by other vehicles, wrong visual features are used within the network, and this affects the whole topology of the lanes.

Second, although historical frames are widely used for feature aggregation, the topological structure of the lanes is seldom restricted in time. This leads to jittery prediction of the lane structure from frame to frame. Third, typical optimization approaches depend almost exclusively on loss functions at the pixel level or row level. The global properties of roads, like smooth curvature and consistent lane distance, are not taken into consideration during training. This leads to the prediction of physically impossible or very rough lane boundaries in practical situations. Hence, there is a need for developing a lane detection method that takes into consideration uncertainty of features, temporal stability, and physical road geometry.

In order to deal with these difficulties, a spatio-temporal graph learning approach is proposed in this paper, as conceptually illustrated in Figure 1. In particular, spatial appearance and temporal context are utilized together for modeling lane geometry. First, a confidence-guided dynamic graph construction component is designed, in which graph connectivity is learned from pairwise node features and refined using uncertainty-aware confidence weighting and Top-*k* sparsification. This prevents high-variance nodes from being considered during heavy occlusion. Second, a temporal consistency scheme is proposed to make the learned adjacency matrix consistent across time steps and thus avoid the structure from being changed abruptly.

**Figure 1:**
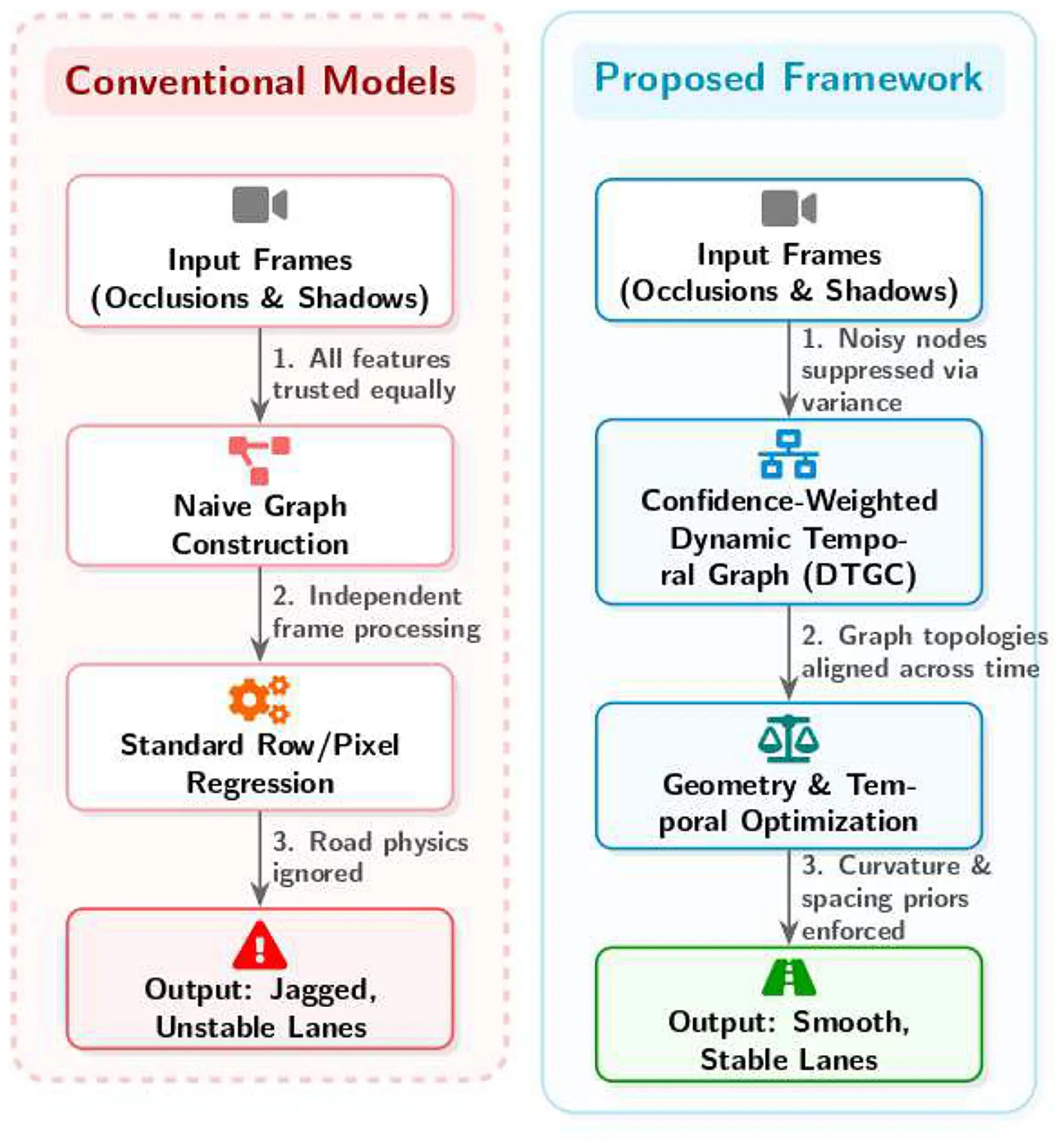
A conceptual comparison between conventional graph-based lane detection and the proposed framework. The three primary research gaps (feature noise propagation, temporal instability, and lack of physical constraints) are directly addressed by the proposed uncertainty weighting, temporal consistency, and geometry-aware optimization modules.

The contributions of this work are summarized as follows:

1. A unified spatio-temporal graph learning framework is proposed by integrating spatial feature extraction, temporal feature aggregation, dynamic graph construction, graph attention-based reasoning, uncertainty estimation, and confidence prediction within a single end-to-end architecture for lane detection.
2. A row-aware graph representation is introduced, in which lane keypoints are represented as graph nodes and their relationships are dynamically modeled through graph attention to capture the structural characteristics of lane boundaries.
3. A confidence- and uncertainty-guided lane prediction mechanism is developed, enabling simultaneous estimation of lane coordinates, prediction confidence, and uncertainty to improve the reliability of lane detection.
4. A multi-objective optimization strategy is designed by jointly optimizing lane coordinate regression, confidence estimation, uncertainty-aware heteroscedastic loss, temporal consistency, and geometry-aware constraints to produce accurate and structurally coherent lane predictions.

The rest of the paper is organized as follows. Section 2 presents the spatio-temporal graph learning framework that was proposed, along with the details on the dynamic graph and geometry-aware optimizations. The experiment settings and datasets used in the experiments are discussed in Section 3. The results of the experiments, the ablation study, and comparison with internal baselines are presented in Section 6.

## 2 Related works

Lane detection has advanced from standard CNN based segmentation approaches to anchor-based localization, transformer-based global context modeling, spatio-temporal learning, and geometry-aware optimization approaches. Recently, there have been many advancements in terms of localization accuracy, efficiency, and robustness under difficult driving conditions. However, current approaches usually concentrate on either one or two facets of lane perception, but relatively less effort has been made for developing models which simultaneously model temporal dynamics, adaptive structure, uncertainty, and geometry-aware optimization. This paper reviews the related literature in four different perspectives considering the current research trends.

### 2.1 Anchor-based spatial lane detection

Recent advancements in anchor-based lane detection systems have greatly enhanced localization efficiency through the use of attention, multi-scale feature improvement, and anchor-based classification (Zheng et al., 2022; Tabelini et al., 2021). LaneATT model utilized an anchor-based attention network to combine global context for efficient localization of lanes (Tabelini et al., 2021). CLRNet further enhanced localization accuracy using cross-layer refinement which incorporates high-level semantic features with low-level spatial features while improving feature representation with ROIGather (Zheng et al., 2022). UFLDv2 was proposed as a novel anchor-based ordinal classification problem for lane detection to achieve remarkable inference efficiency while incorporating global context for efficient localization of coordinates (Qin et al., 2024). In spite of these advancements, these models are inherently designed as single-frame approaches and hence fail to leverage the temporal coherence across consecutive frames. Furthermore, even though these approaches employ different attention and context fusion strategies, they fail to explicitly model the prediction uncertainty that could be leveraged to control feature interaction and dynamic creation of graph dependencies between lane keypoints. Consequently, feature interactions remain appearance-driven and are more susceptible to occlusions, illumination variations, and noisy observations.

### 2.2 Transformer-based lane representation and global context learning

One of the earliest transformer-based approaches for lane detection is the Laneformer by Han et al. (2022), which utilizes the row-column self-attention and object-aware attention to model the long-range dependencies between lanes and the surrounding vehicles. Thereby circumventing the localization restrictions of the CNN-based approaches. Nonetheless, the Laneformer mainly concerns the modeling of spatial relationships within an individual image and uses attention maps without considering the temporal consistency and lane connectivity. Chen et al. (2022) proposed PersFormer, which leveraged the transformer-based representation and developed the PersFormer for the 3D lane detection based on the Perspective Transformer, which enables the learning of feature transformation from the front-view image to bird's-eye-view (BEV) space and performs the joint optimization of 2D and 3D lane detection. Nevertheless, the attention strategy of the PersFormer still focuses on the views and does not model the interactions between lane keypoints and uncertainty estimation. More recently, LDTR is presented by Yang et al. (2024), in which the problem of lane detection is redefined with the help of anchor-chain formulation and multi-referenced deformable attention in order to handle complex lane geometries without any form of hand-crafted post-processing. Although LDTR significantly improves global contextual representation, it does not explicitly incorporate uncertainty-aware feature weighting or adaptive graph reasoning for modeling structural relationships among lane keypoints. Overall, transformer-based lane detectors substantially improve long-range contextual modeling and lane representation; however, they still lack a unified mechanism for jointly modeling temporal evolution, uncertainty-aware feature learning, and adaptive graph-based relational reasoning.

### 2.3 Spatio-temporal lane modeling

In a study, Dong et al. (2023) presented a hybrid spatial-temporal deep learning network architecture combining SCNN with ConvLSTM/ConvGRU by taking advantage of temporal coherence from frame to frame. Although temporal modeling improves robustness over single-frame approaches, the reliance on CNN and recurrent networks limits its ability to capture long-range dependencies and complex lane geometries. Bai et al. (2023) proposed CurveFormer. This method uses transformer networks for formulating 3D lane detection through dynamic curve queries and curve cross-attention without using the bird's eye view (BEV) transformation process. Although CurveFormer effectively models global context, it primarily focuses on sparse query refinement for 3D lane estimation. In a more recent study, Yang et al. (2024) presented LDTR. This utilizes anchor-chain representation, along with multi-referenced deformable attention, for improving the representation of lanes. Collectively, these methods improve temporal and geometric lane modeling; however, adaptive graph construction and uncertainty-guided relational reasoning remain largely unexplored.

### 2.4 Geometry-aware and anchor-based lane optimization

Huang et al. (2023) presented Anchor3DLane, which is a framework for monocular 3D lane detection without BEV, which allows the direct regression of 3D lane anchors from front-view features through anchor projection and iterative regression. Similarly, Qin et al. (2020) proposed UFLDv2, which formulates lane detection as a hybrid row-column anchor-based ordinal classification problem. Anchor3DLane aims at accurate estimation of 3D lane geometry via spatial anchor regression, while UFLDv2 aims at fast 2D lane localization using hybrid sparse anchors and ordinal classification-based coordinates. Anchor3DLane depends upon the pre-defined anchor distributions and camera calibration, whereas the iterative regression and hand-crafted geometric optimization in the algorithm do not incorporate any adaptation for the lane relationship or uncertainties in the features. On the other hand, UFLDv2 performs independent learning of lane locations using fixed classification of the anchors without incorporating any dependency between the lanes and temporal coherence between subsequent frames. Consequently, neither method explicitly models adaptive lane relationships, uncertainty-aware feature interactions, or spatio-temporal graph reasoning. Even though previous works have made notable contributions to lane detection from anchor-based localization to transformer-based representation learning, spatio-temporal modeling, and geometry-aware optimization, such methods only enhance certain aspects of lane detection but not all of them simultaneously. Table 1 presents the main drawbacks of the representative research directions, and shows how the proposed GraphLane tackles these problems with Dynamic Temporal Graph Construction (DTGC), uncertainty-aware graph reasoning, temporal feature aggregation, and geometry-aware optimization. Despite significant advancements made via anchor-based localization, Transformer network models, temporal learning, and geometry-aware optimization, the above modules have been mostly studied separately from each other. The existing solutions usually lack the joint use of dynamic temporal graph construction, uncertainty-guided relational reasoning, temporal feature propagation, and geometry-aware optimization in an integrated manner. Motivated by these limitations, the proposed GraphLane framework integrates temporal feature aggregation, Dynamic Temporal Graph Construction (DTGC), confidence-weighted graph reasoning, and geometry-aware optimization into a unified end-to-end architecture. The overall framework is presented in the following section.

**Table 1:** Comparison of existing lane detection research directions and the key contributions of the proposed GraphLane framework.

Research category	Common limitation	How GraphLane addresses it
Anchor-based spatial methods (LaneATT, CLRNet, UFLDv2)	Process each frame independently without exploiting temporal continuity. They also lack explicit uncertainty modeling and adaptive structural reasoning.	Integrates temporal context from consecutive frames using a Transformer encoder and performs Dynamic Temporal Graph Construction (DTGC) for adaptive structural reasoning.
Transformer-based methods (LaneFormer, PersFormer, LDTR)	Capture global context effectively but rely on frame-wise attention and do not explicitly model graph topology or prediction uncertainty.	Constructs adaptive lane graphs through DTGC and suppresses unreliable node interactions using uncertainty-guided confidence weighting before graph reasoning.
Spatio-temporal methods (Hybrid CNN-LSTM, CurveFormer)	Utilize temporal information but do not explicitly learn structural relationships among lane keypoints or maintain graph consistency.	Combines temporal feature aggregation with graph attention reasoning and incorporates a temporal graph consistency loss for stable lane localization.
Geometry-aware optimization (Anchor3DLane, UFLDv2)	Employ geometric constraints or anchor optimization independently without integrating adaptive graph learning and uncertainty estimation.	Employs a unified multi-objective optimization consisting of coordinate regression, geometry-aware constraints, uncertainty-aware heteroscedastic loss, and temporal graph consistency.

## 3 Methodology

The localization of lanes using autonomous lane detection needs the concurrent extraction of spatial characteristics, temporal consistency, and the relation between different keypoints of lanes under varied driving scenarios. Current convolutional based methods use only the local image feature information, whereas most of the graph-based methods use fixed graph structure which is unable to adapt to the changes in scenes. In order to overcome these issues, the GraphLane framework has been proposed, which combines spatial feature extraction, temporal context aggregation, graph construction, and graph attention reasoning into a single end-to-end pipeline.

### 3.1 Overall framework

The architectural flow of the proposed lane detection pipeline is shown in Figure 2. This lane detector model is designed to integrate spatial appearance, temporal information, and graph-based relational reasoning for lane coordinate localization. For lane detection, an image sequence of three consecutive frames is fed into the proposed model; where the current road image is considered along with two previous frames. The current frame is used to extract the spatial features, while the three frames are considered for temporal context extraction. The spatial features are extracted using a light-weight convolutional backbone that includes convolutional blocks and residual blocks. Similarly, the same backbone is used separately for each temporal frame. Then, temporal features are combined using transformer encoder for the generation of a compact representation of the historical image scene. The generated spatial and temporal features are further projected into a common feature space known as graph feature space. Graph structure is formed using learnable node embeddings, learnable row embeddings, and the projected spatial-temporal features. In the graph, each node represents a fixed lane keypoint position, while the row embeddings represent positional information for row-anchor representation. The graph nodes are initialized for dynamic graph formation. First, an uncertainty estimation module predicts a variance value for each graph node, which is subsequently converted into confidence scores through an exponential transformation. These confidence values are incorporated during graph construction to suppress unreliable node interactions. Pairwise node features are processed by a multilayer perceptron to estimate adaptive edge weights, which are subsequently refined through confidence weighting, Top-*k* sparsification, graph symmetrization, and self-loop addition to obtain the final adjacency matrix. The constructed graph is used to perform graph reasoning with two graph attention layers. After applying the first graph attention layer, the node features are assigned based on the confidence and then re-estimated node uncertainty prior to the second graph attention layer.

**Figure 2:**
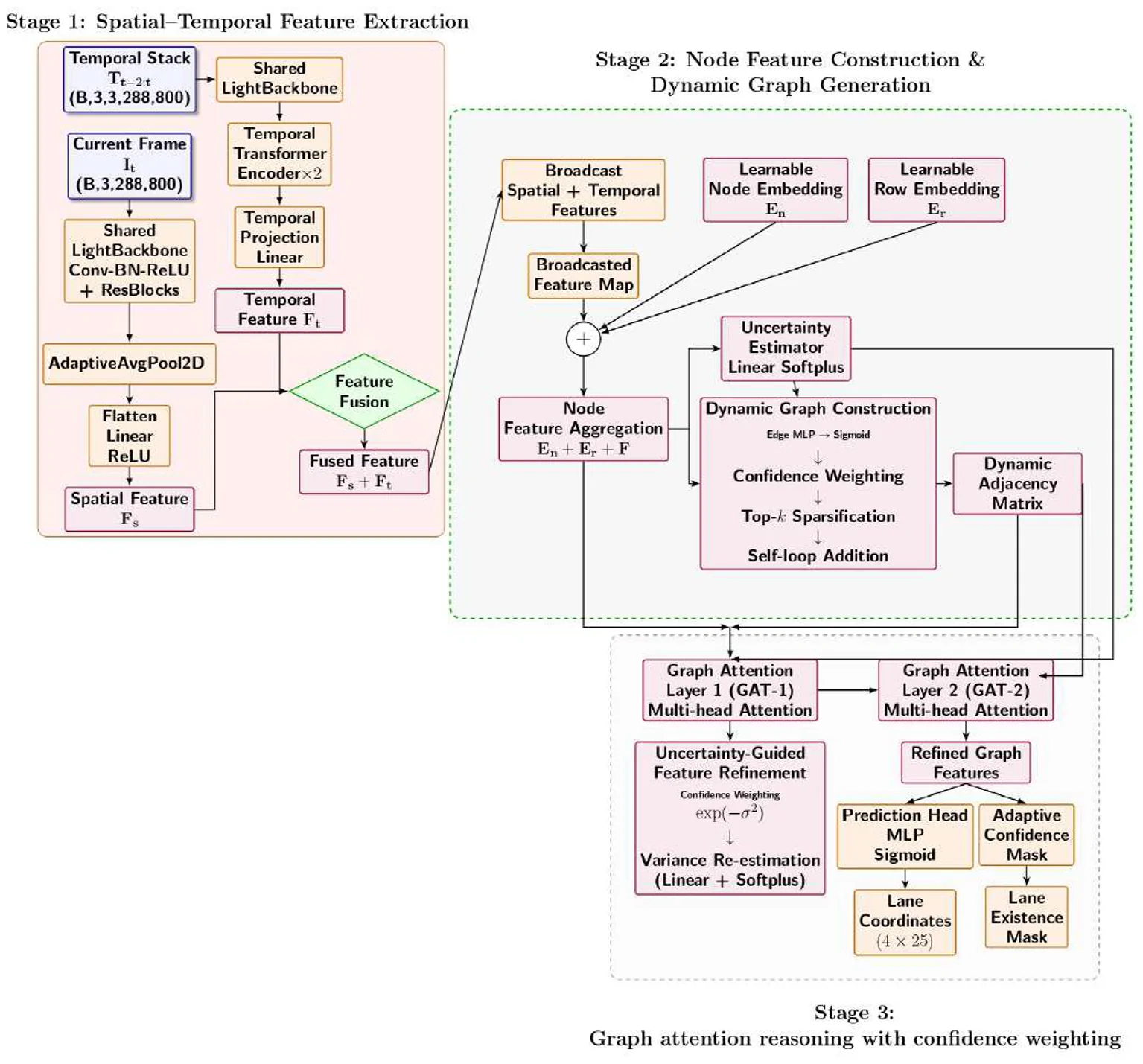
Overall architecture of the proposed GraphLane framework. The framework consists of three stages: (1) spatial-temporal feature extraction, (2) node feature construction and dynamic graph generation, (3) graph attention reasoning with confidence weighting for lane coordinate localization and lane existence prediction.

Finally, the obtained node features are used by two prediction branches. One of the branches estimates normalized lane coordinates for all predefined row-anchors, while the other branch predicts the confidence for each lane keypoint. During the training stage, these predicted lane coordinates and confidence values are optimized in a joint manner with a composite loss function that consists of coordinate regression, geometry, confidence supervision, uncertainty-aware heteroscedastic loss, and temporal graph consistency loss terms. Let the current input image be denoted by *I*_*t*_, the temporal image sequence by $T=\left\{I_{t-2},I_{t-1},I_{t}\right\}$, and the predicted lane coordinates by $\hat{\text{Y}}$. The overall mapping performed by the model can be expressed as Equation 1:


$$\begin{array}{l} \hat{\text{Y}},\hat{\text{C}}=f\left(I_{t},T;\Theta \right) \end{array}$$ (1)

where *f*(·) denotes the proposed network, $\hat{\text{Y}}$ represents the predicted lane coordinates, $\hat{\text{C}}$ denotes the predicted confidence scores, and Θ represents the learnable network parameters.

The entire flow for the presented framework is described through Algorithm 1. This algorithm covers the entire procedure of forward propagation, including the spatial feature extraction, temporal context integration, graph building, graph reasoning, and lane prediction. The processing stages presented in Algorithm 1 are explained in detail in Sections 3.2–3.9.

Algorithm 1 Training procedure of the proposed GraphLane framework. 
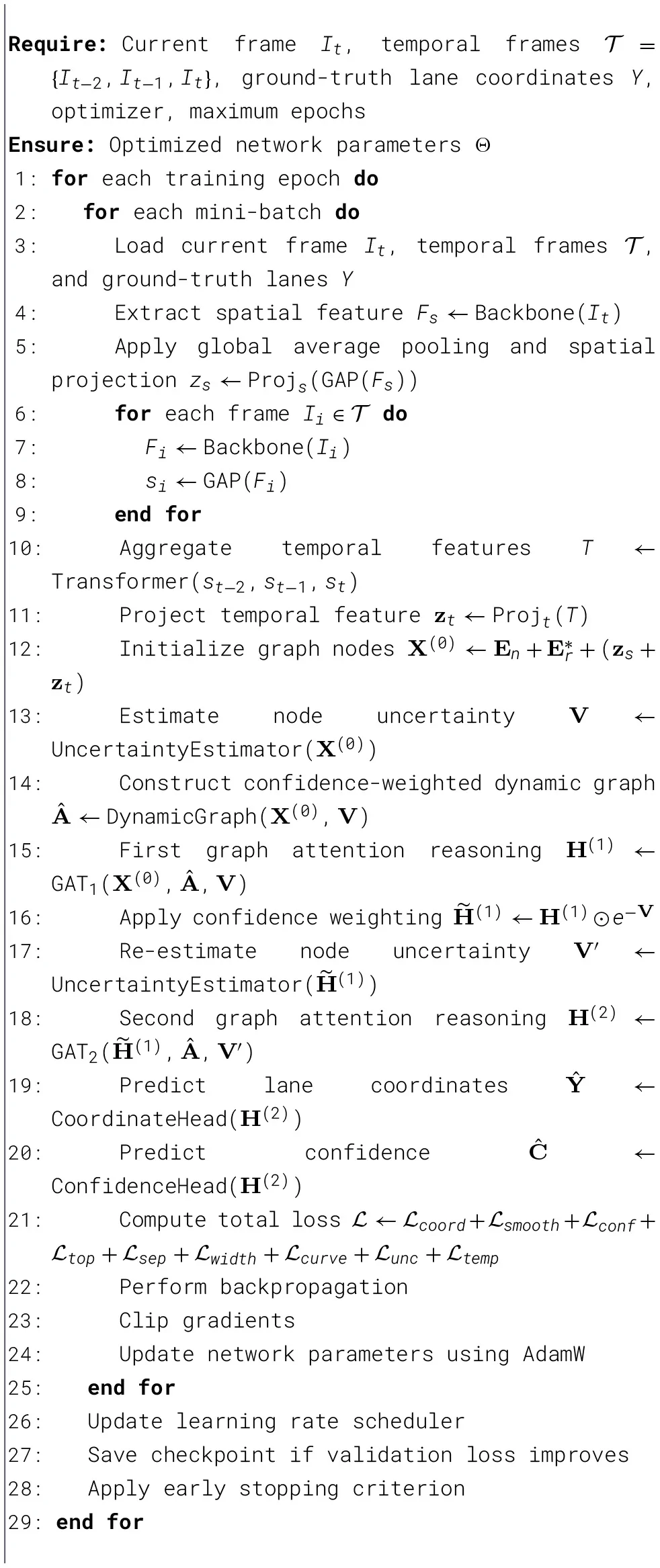

### 3.2 Spatial feature extraction

The input image from the current dataset was initially analyzed to acquire its spatial appearance features through a lightweight convolutional backbone. The convolutional backbone was built by combining an initial convolutional block with a number of residual feature extraction blocks. Every convolutional block involved a convolution process, batch normalization, and a rectified linear unit (ReLU) function. Residual links were added to the backbone network to help feature propagation and to make training easier. The backbone was gradually widening its receptive field while retaining the structural information necessary for lane detection. Let the current input image be denoted as $I_{t}\in {\mathbb{R}}^{H\times W\times 3}$. The spatial feature extraction process can be expressed as Equation 2:


$$\begin{array}{l} {\text{F}}_{s}=B\left(I_{t}\right), \end{array}$$ (2)

where *B*(·) denotes the convolutional backbone and **F**_*s*_ represents the extracted spatial feature map. A pooling operation on the final feature map is used to generate a compact feature representation for constructing a graph. Then, the pooled feature vector was embedded in the graph feature space using a fully connected layer. The projection of convolutional features to a fixed dimension representation which is compatible with the graph reasoning model is performed using the Equation 3.


$$\begin{array}{l} {\text{z}}_{s}=W_{s}\left(\mathrm{GAP}\left({\text{F}}_{s}\right)\right)+b_{s}, \end{array}$$ (3)

where GAP(·) denotes the global average pooling operation, *W*_*s*_ and *b*_*s*_ represent the learnable projection parameters, and ${\text{z}}_{s}\in {\mathbb{R}}^{d}$ is the projected spatial feature vector with feature dimension *d* = 128. Afterwards, the projected spatial feature vector was broadcast to all graph nodes during graph node initialization. As a result, each node initially receives the same global spatial context before node-specific structural conditioning and graph reasoning are applied. Graph-specific knowledge was added later on during graph construction and graph attention steps. Node-specific structural information is introduced through the learnable node and row embeddings during graph initialization and is subsequently refined through dynamic graph construction and graph attention.

### 3.3 Temporal context aggregation

Temporal information can be helpful for detecting lanes in dynamic driving scenarios due to the nature of the information that comes from consecutive frames. Hence, the temporal context was included through the creation of a sequence containing the current frame and the previous two frames. All frames were independently processed using the same convolutional backbone used to extract spatial features. This allowed keeping all frames in the same feature space without extra parameters for the model. Let the temporal image sequence be denoted as Equation 4


$$\begin{array}{l} T=\left\{I_{t-2},I_{t-1},I_{t}\right\}. \end{array}$$ (4)

Each frame in the sequence was first processed by the convolutional backbone to obtain its corresponding feature representation as in Equation 5,


$$\begin{array}{l} {\text{F}}_{i}=B\left(I_{i}\right),\;I_{i}\in T, \end{array}$$ (5)

where *B*(·) denotes the shared backbone network.

The extracted feature maps were subsequently transformed into compact feature vectors through global average pooling. The pooled features from all temporal frames were stacked to form a temporal feature sequence as in Equation 6,


$$\begin{array}{l} \text{S}=\left[{\text{s}}_{t-2},{\text{s}}_{t-1},{\text{s}}_{t}\right], \end{array}$$ (6)

where each feature vector was obtained as in Equation 7


$$\begin{array}{l} {\text{s}}_{i}=\mathrm{GAP}\left({\text{F}}_{i}\right). \end{array}$$ (7)

The temporal feature sequence was then supplied to a transformer encoder (Vaswani et al., 2017) consisting of two encoder layers with eight attention heads. Self-attention was employed to capture the relationships among consecutive frames and aggregate temporal information into a unified representation. The temporal aggregation process is expressed as Equation 8


$$\begin{array}{l} \text{T}=\mathrm{Transformer}\left(\text{S}\right), \end{array}$$ (8)

where **T** denotes the aggregated temporal representation.

The transformer output was projected into the graph feature space using a linear projection layer, **z**_*t*_ = *W*_*t*_**T**+*b*_*t*_, where *W*_*t*_ and *b*_*t*_ represent the learnable projection parameters. The proposed temporal feature vector was of the same dimensional size as the spatial feature representation; hence, both could be combined when initializing the graph nodes. As opposed to the spatial feature that was extracted from just the current frame, the temporal feature encoded the context from three consecutive frames. This temporal representation was subsequently fused with the spatial representation and used as part of the graph node initialization.

### 3.4 Graph node construction

Graph reasoning works on a pre-defined set of graph nodes, in which each node corresponds to a particular lane keypoint. The projection of spatial features and temporal features were combined with the node embeddings and row embeddings in order to construct the graph-based representation. This architecture allows each node to capture global scene information, temporal information, node information, and positional information in terms of row-anchor representation.

Firstly, the projected spatial feature vector computed from the current image frame and the projected temporal feature vector from the transformer encoder were fused through element-wise addition to obtain a unified scene representation,


$$\begin{array}{l} \text{z}={\text{z}}_{s}+{\text{z}}_{t}, \end{array}$$ (9)

where *z*_*s*_ and *z*_*t*_ denote the projected spatial and temporal feature vectors, respectively. Since the resulting scene representation captures global spatial-temporal context rather than information specific to individual lane keypoints, the fused feature vector is broadcast along the node dimension so that every graph node receives the same global scene context before graph reasoning.

Two complementary learnable embeddings are introduced to provide structural identity to the graph nodes. The first embedding represents the identity of each lane-keypoint node, whereas the second embedding represents the predefined row-anchor position. Let *E*_*n*_ = [*e*_1_, *e*_2_, …, *e*_*N*_] denote the learnable node-identity embeddings, where *N* = *L*×*K* is the total number of lane-keypoint nodes, with *L* denoting the number of lanes and *K* the number of row anchors. Let *E*_*r*_ = [*r*_1_, *r*_2_, …, *r*_*K*_] denote the learnable row embeddings, which are shared across lane identities but associated with their corresponding row anchors.

The initialized representation of node *i* = (*l, k*), corresponding to lane identity *l* and row anchor *k*, can therefore be written as Equations 9, 10


$$\begin{array}{l} {\text{x}}_{l,k}^{\left(0\right)}={\text{e}}_{l,k}^{node}+{\text{r}}_{k}+\text{z}, \end{array}$$ (10)

Equivalently, the complete node feature matrix is given as Equation 11


$$\begin{array}{l} X^{\left(0\right)}=E_{n}+E_{r}^{*}+1_{N}{\text{z}}^{⊤}, \end{array}$$ (11)

where $E_{r}^{*}\in {\mathbb{R}}^{N\times d}$ denotes the row embeddings expanded across the lane identities. Although the fused spatial-temporal representation **z** is shared across all nodes, the resulting node representations are not identical because each node is associated with a distinct lane-keypoint identity and a specific row-anchor representation. Thus, the broadcast operation provides a common visual context rather than a common node representation.

This formulation is an important design distinction in GraphLane. The network does not attempt to retain an independent cropped image feature for every lane keypoint after global average pooling. Instead, the globally pooled spatial and temporal representations provide a shared description of the driving scene, while the learnable node and row embeddings condition this shared evidence according to the lane-row hypothesis represented by each node. In this sense, GraphLane decouples global visual evidence from node-specific structural interpretation.

The node-conditioned representations are subsequently used by the dynamic graph construction module rather than the global scene vector alone. Pairwise node representations are evaluated to learn scene-dependent edge strengths, and the resulting graph is further processed by graph attention. Consequently, the same visual scene context can be interpreted differently for different lane-keypoint hypotheses according to their node identity, row-anchor position, and learned relationships with neighboring nodes.

This design is particularly relevant for challenging cases in which direct lane appearance is degraded. When a lane marking is partially occluded or visually ambiguous, the corresponding lane-row hypothesis remains explicitly represented in the graph through its node identity and row-anchor embedding. Its representation can then be refined using the temporal scene context and information propagated from dynamically selected neighboring nodes. Therefore, GraphLane does not require direct local visual evidence to be independently stored at every node; instead, it maintains a structured lane-keypoint hypothesis and refines it through adaptive relational reasoning.

Importantly, the shared scene vector does not imply identical node representations, because node identity and row-anchor embeddings are added before graph construction. In other words,


$$\begin{array}{r} \text{Shared visual context}+\text{node identity}+\text{row-anchor identity}\\ \to \text{node-conditioned representation} \end{array}$$

Figure 3 provides a visual summary of the node-conditioned spatio-temporal reasoning process in GraphLane. The spatial and temporal branches first generate a shared global scene representation, which is broadcast to all graph nodes. Each lane-keypoint node is then differentiated by its learnable node-identity and row-anchor embeddings, producing node-conditioned representations before uncertainty estimation and dynamic graph construction. This illustrates how GraphLane transforms shared visual context into differentiated lane-keypoint hypotheses without requiring an independent local image feature to be retained for every node. As illustrated in Figure 3, GraphLane does not obtain node-specific visual evidence by extracting separate image regions around individual lane keypoints. Instead, the spatial and temporal branches provide a shared description of the driving scene, while the node-identity and row-anchor embeddings specify the lane-keypoint hypothesis to which this shared evidence is assigned. The resulting differentiated node representations are then used to estimate uncertainty and construct scene-dependent graph relationships. Graph attention subsequently propagates information among the selected lane-keypoint nodes, producing refined node states for coordinate and confidence prediction. This design allows the same global visual context to be interpreted differently for different lane-row hypotheses while maintaining a structured representation for keypoints whose direct lane appearance may be degraded by occlusion.

**Figure 3:**
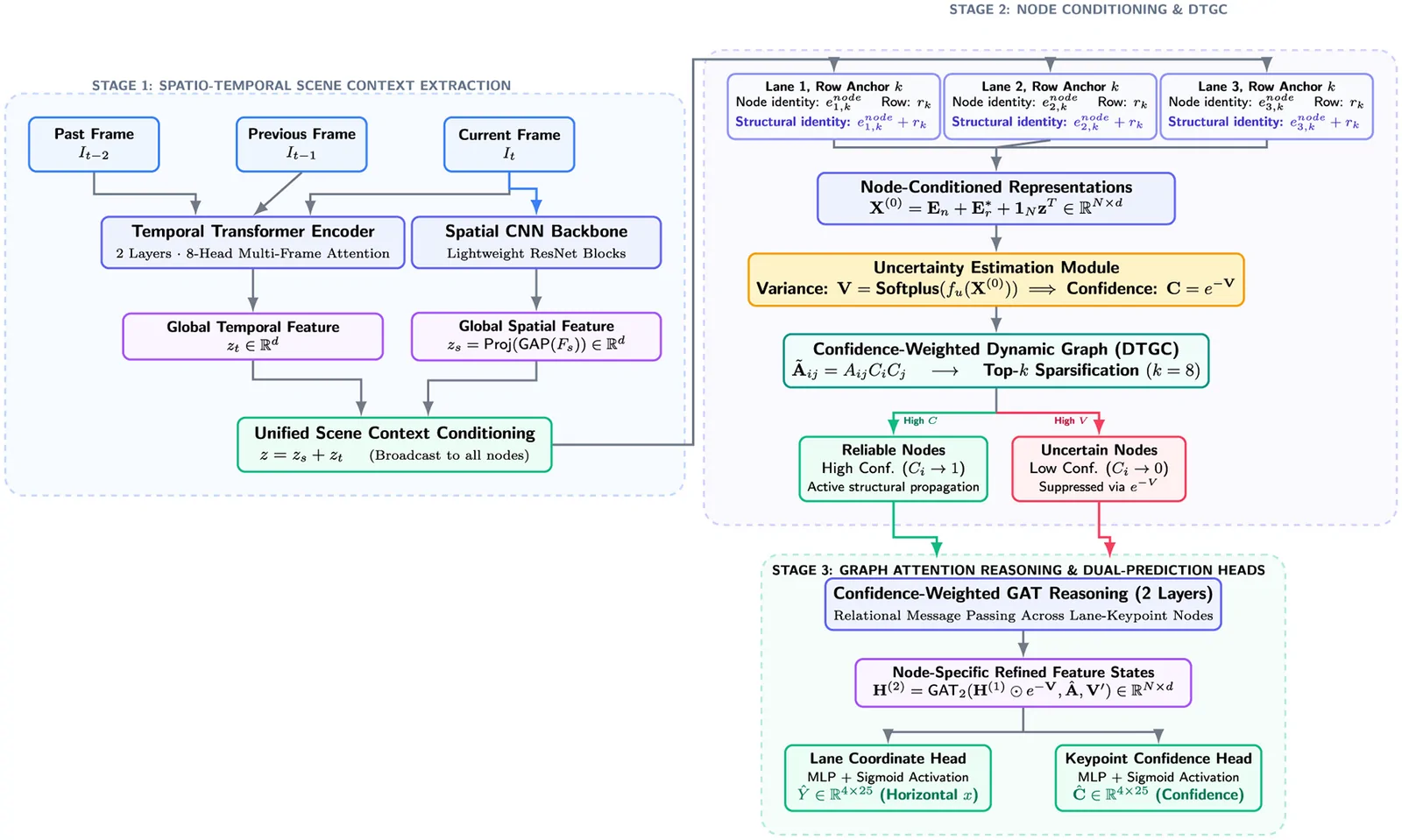
Node-conditioned spatio-temporal reasoning in GraphLane. The spatial and temporal branches generate a shared global scene representation that is broadcast to all lane-keypoint nodes. Distinct learnable node-identity and row-anchor embeddings condition the shared representation to form differentiated node states. These node-conditioned representations are subsequently processed through uncertainty estimation, confidence-weighted Dynamic Temporal Graph Construction (DTGC), and graph attention reasoning to produce node-specific lane coordinates and keypoint confidence predictions.

### 3.5 Dynamic graph construction with confidence weighting

Graph topology was generated adaptively for each input instance by leveraging the initialized features of the nodes. The graph topology was not generated based on any preset graph but through learning its connections from the features of the nodes dynamically. In addition, node uncertainty was taken into account while generating the graph topology. Figure 4 gives a conceptual comparison of traditional dynamic graph construction techniques and the proposed Dynamic Temporal Graph Construction (DTGC). While conventional techniques construct graphs on the basis of pre-defined adjacency or similarity, the proposed DTGC technique uses uncertainty-driven edge learning and Top-*k* sparsification to create sparse and adaptive graphs before graph reasoning.

**Figure 4:**
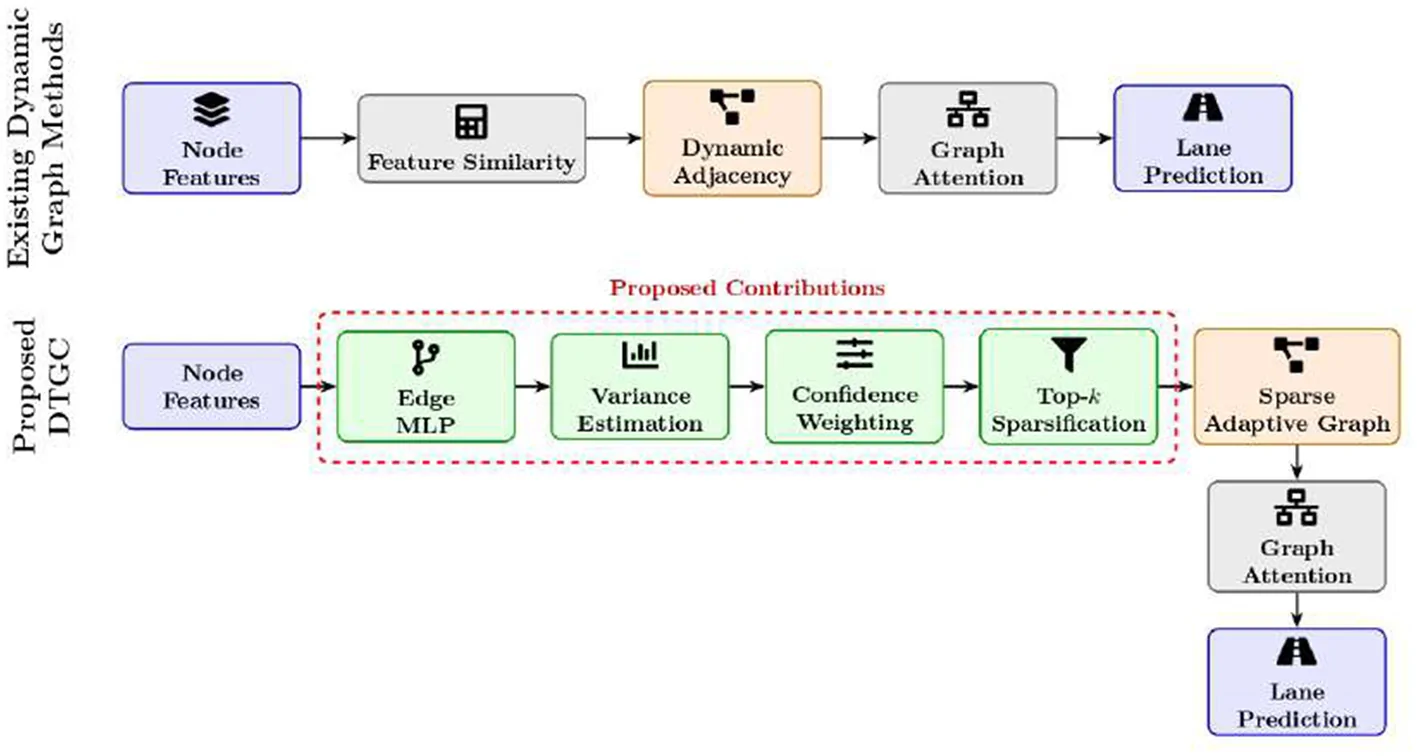
Methodological comparison between conventional dynamic graph construction and the proposed DTGC. Unlike existing methods, DTGC learns confidence-weighted sparse graph connectivity through Edge MLP, variance estimation, and Top-*k* sparsification.

Because the initialized nodes contain both the shared scene representation and node-specific structural embeddings, dynamic graph construction operates on differentiated lane-keypoint hypotheses rather than on identical copies of the global visual vector. The learned pairwise relationships therefore determine which lane-keypoint hypotheses should exchange information for the current scene. This provides the transition from shared visual context to adaptive node-level relational reasoning.

Let the initialized graph node feature matrix defined in Section 3.4 be denoted by Equation 12:


$$\begin{array}{l} {\text{X}}^{\left(0\right)}=\left[{\text{x}}_{1}^{\left(0\right)},{\text{x}}_{2}^{\left(0\right)},\ldots ,{\text{x}}_{N}^{\left(0\right)}\right], \end{array}$$ (12)

where *N* denotes the total number of graph nodes.

- Variance estimation: the uncertainties linked to each graph node were estimated in a first step via a lightweight multilayer perceptron with Softplus activation function for ensuring non-negative variance estimates. The uncertainty estimation takes the form of Equation 13.
$$\begin{array}{l} \text{V}=\mathrm{Softplus}\left(f_{u}\left({\text{X}}^{\left(0\right)}\right)\right), \end{array}$$ (13)
  where *f*_*u*_(·) denotes the uncertainty estimation network and **V** represents the estimated node variance. The estimated variance was subsequently converted into node confidence values using the exponential transformation, **C** = exp(−**V**), where larger uncertainty values correspond to lower node confidence. The Softplus activation ensures non-negative variance estimates while maintaining stable gradient propagation during training. These confidence values reduce the contribution of highly uncertain node interactions during graph construction without introducing additional learnable parameters.
- Dynamic edge weight estimation: to estimate the relationship between graph nodes, pairwise node features were first constructed by concatenating the feature vectors of every node pair represented in Equation 14,
$$\begin{array}{l} {\text{e}}_{ij}=\left[{\text{x}}_{i}^{\left(0\right)}∥{\text{x}}_{j}^{\left(0\right)}\right], \end{array}$$ (14)
  where ∥ denotes the concatenation operator. The concatenated features were supplied to a multilayer perceptron to estimate the pairwise edge weights using Equation 15,
$$\begin{array}{l} A_{ij}=\sigma \left(f_{e}\left({\text{e}}_{ij}\right)\right), \end{array}$$ (15)
  where *f*_*e*_(·) denotes the edge prediction network and σ(·) is the sigmoid activation function.
- Confidence-weighted graph construction: the estimated edge weights were subsequently modulated using the confidence values of the corresponding node pair by Equation 16,
$$\begin{array}{l} {Ã}_{ij}=A_{ij}C_{i}C_{j}, \end{array}$$ (16)
  where *C*_*i*_ and *C*_*j*_ denote the confidence values of nodes *i* and *j*, respectively. In order to simplify the graph, only the strong neighbor relations were selected for each node. In particular, the top-k edge strengths were kept, whereas the rest were dropped. The resulting graph was symmetrized using the average of the adjacency matrix with its transpose as in Equation 17,
$$\begin{array}{l} \text{A}=\frac{\tilde{\text{A}}+{\tilde{\text{A}}}^{T}}{2}. \end{array}$$ (17)
  Finally, self-loops were added to every graph node by incorporating the identity matrix represented as Equation 18,
$$\begin{array}{l} \hat{\text{A}}=\text{A}+\text{I}, \end{array}$$ (18)
  where **I** denotes the identity matrix.
  The resulting adjacency matrix was subsequently supplied to the graph attention module for relational reasoning.
  Unlike the existing dynamic graph learning paradigm where graph connectivity is constructed based on pre-defined adjacency matrix or feature similarity, the proposed method constructs the graph structure by performing the following three consecutive steps. The first step involves learning of pairwise node relations based on the Edge MLP network, such that graph connectivity can be updated according to the current scene instead of being defined by hand-designed neighborhood structure. The second step modulates the learned edge weights using node confidence derived from the estimated uncertainty, thereby reducing the influence of unreliable node interactions before graph attention reasoning. Finally, the Top-*k* sparsification selects the most important graph connections for generating sparse and discriminative graph structure. Consequently, the proposed DTGC simultaneously adapts graph topology, suppresses unreliable feature interactions, and maintains computational efficiency for lane localization.

### 3.6 Confidence-weighted graph attention reasoning

Following the construction of the dynamic graph, graph reasoning was applied to propagate information between the neighboring keypoints of the lanes. This propagation was done using two graph attention layers that updated the representation of the nodes according to the graph structure obtained from the graph learning process. Between the two graph attention layers, feature weighting and uncertainty re-estimation based on confidence was carried out. Let the initialized node features and the dynamically constructed adjacency matrix be denoted by **X** and $\hat{\text{A}}$, respectively.

- First graph attention layer: the initialized node features were first processed by the graph attention layer as in Equation 19,
$$\begin{array}{l} {\text{H}}^{\left(1\right)}={\mathrm{GAT}}_{1}\left(\text{X},\hat{\text{A}},\text{V}\right), \end{array}$$ (19)
  where **V** denotes the uncertainty estimated during graph construction. The graph attention layer aggregates information from neighboring nodes according to the learned adjacency matrix, producing refined node representations while preserving the graph connectivity.
- Confidence-based feature weighting: Following the first graph attention layer, the refined node features were weighted using the confidence values derived from the estimated uncertainty. The confidence weighting process is expressed as Equation 20
$$\begin{array}{l} {\text{H}}^{\left(1\right)}={\text{H}}^{\left(1\right)}⊙\exp\left(-\text{V}\right), \end{array}$$ (20)
  where ⊙ denotes element-wise multiplication. Since the confidence values lie in the interval (0, 1], this operation suppresses unreliable node representations while preserving highly confident features.
  This operation reduces the contribution of node features associated with higher uncertainty while preserving information from more reliable graph nodes.
- Uncertainty re-estimation: since the node representations are updated after graph attention, the uncertainty associated with each node may also change. Therefore, node uncertainty was re-estimated using the updated node features (Equation 21),
$$\begin{array}{l} {\text{V}}^{\prime }=\mathrm{Softplus}\left(f_{u}\left({\text{H}}^{\left(1\right)}\right)\right), \end{array}$$ (21)
  where *f*_*u*_(·) denotes the uncertainty estimation network.
  The re-estimated uncertainty was subsequently supplied to the second graph attention layer.
- Second graph attention layer: the confidence-weighted node features were further refined using a second graph attention layer as Equation 22,
$$\begin{array}{l} {\text{H}}^{\left(2\right)}={\mathrm{GAT}}_{2}\left({\text{H}}^{\left(1\right)},\hat{\text{A}},{\text{V}}^{\prime }\right), \end{array}$$ (22)
  where **H**^(2)^ represents the final graph representation. The output nodes include information regarding the global environment, temporal information, positional encodings, and relational information that is carried through using the dynamically created graph. These processed node features are then passed on to the lanes prediction and confidence prediction paths.

### 3.7 Lane coordinate prediction

The graph nodes generated through the graph attention reasoning process were fed into the coordinate prediction head to predict the lane geometry. The coordinate prediction head used a multilayer perceptron that independently regressed the horizontal coordinate for each of the predefined lane keypoints. As the row-anchor coordinates were constant when preparing the training data, only the horizontal coordinates had to be predicted. Let the refined graph node features be denoted by Equation 23


$$\begin{array}{l} {\text{H}}^{\left(2\right)}=\left[{\text{h}}_{1},{\text{h}}_{2},\ldots ,{\text{h}}_{N}\right], \end{array}$$ (23)

where *N* represents the total number of graph nodes. The coordinate prediction head transforms these node features into normalized lane coordinates (Equation 24),


$$\begin{array}{l} \hat{\text{Y}}=f_{p}\left({\text{H}}^{\left(2\right)}\right), \end{array}$$ (24)

where *f*_*p*_(·) denotes the coordinate prediction network and $\hat{\text{Y}}$ represents the predicted lane coordinates. To ensure that the predicted coordinates remain within the image boundaries, a sigmoid activation function was applied at the output layer as illustrated in Equation 25,


$$\begin{array}{l} \hat{x}=\sigma \left(z\right), \end{array}$$ (25)

where *z* denotes the predicted coordinate before activation and σ(·) is the sigmoid function. Consequently, every predicted coordinate was normalized to the interval $0\leq \hat{x}\leq 1$.

These normalized coordinates were later converted into pixel values by multiplying them by the image width while visualizing and evaluating them quantitatively. As the row-anchor coordinates were fixed, the prediction of a lane was represented by a set of horizontal coordinates representing the row-anchor coordinates.

The lane coordinate prediction branch predicts the geometry of all the predefined lanes at once. This implies that the output can be represented as Equation 26


$$\begin{array}{l} \hat{\text{Y}}\in {\mathbb{R}}^{L\times K}, \end{array}$$ (26)

where *L* = 4 denotes the maximum number of predicted lanes and *K* = 25 denotes the number of row-anchor keypoints associated with each lane. The predicted lane coordinates are subsequently optimized using the coordinate regression and geometry-aware loss functions described in Section 3.9.

### 3.8 Confidence prediction

Besides lane coordinate regression, the proposed framework also predicts confidence for each lane keypoint. The inclusion of this auxiliary prediction stream allows the network to estimate the reliability of individual lane-keypoint predictions and thereby indicate the confidence associated with the predicted lane coordinates. The confidence prediction branch operates on the refined graph representation after the second graph attention layer. The refined node features incorporate the shared spatial-temporal scene context together with node-specific structural conditioning and information propagated through the dynamically constructed graph. The confidence prediction module therefore estimates lane-keypoint reliability from the graph-refined representation rather than directly from the globally pooled visual feature.

The refined graph node features are independently processed by the confidence prediction network to estimate the confidence of each lane keypoint, as expressed in Equation 27,


$$\begin{array}{l} \hat{\text{C}}=f_{c}\left({\text{H}}^{\left(2\right)}\right), \end{array}$$ (27)

where *f*_*c*_(·) denotes a shared multilayer perceptron applied independently to each refined node feature, and $\hat{\text{C}}$ represents the predicted confidence matrix. Since the confidence prediction is performed after graph refinement, each confidence value is conditioned on the corresponding refined node representation, which contains the shared spatial-temporal scene context together with node-specific structural information and information propagated through the learned graph relationships.

To ensure that every confidence value lies within the interval [0, 1], a sigmoid activation function is applied at the output layer, ĉ = σ(*z*), where *z* denotes the predicted confidence before activation. The confidence prediction branch produces one confidence value for every lane keypoint. Consequently, the predicted confidence matrix has the same dimensions as the coordinate prediction output given by Equation 28,


$$\begin{array}{l} \hat{\text{C}}\in {\mathbb{R}}^{L\times K}, \end{array}$$ (28)

where *L* = 4 denotes the maximum number of predicted lanes and *K* = 25 represents the predefined row-anchor positions.

The confidence targets are created directly from the lane annotations present in the ground truth during training. Positive targets are assigned to keypoints that correspond to valid lane coordinates, whereas negative targets are assigned to invalid lane keypoints. This allows the confidence and coordinate prediction branches to be optimized jointly, enabling the network to estimate lane geometry together with the reliability of its individual keypoint predictions.

### 3.9 Geometry-aware optimization

The proposed GraphLane was optimized with a compound objective function, which consisted of the following components: coordinate regression, confidence, geometric regularization, uncertainty-aware heteroscedastic loss, and temporal graph consistency. The objective function is formulated as Equation 29:


$$\begin{array}{l} L_{\text{total}}=\;\lambda_{1}L_{\text{coord}}+\lambda_{2}L_{\text{smooth}}+\lambda_{3}L_{\text{conf}}+\lambda_{4}L_{\text{top}}\\ \;+\lambda_{5}L_{\text{sep}}+\lambda_{6}L_{\text{width}}+\lambda_{7}L_{\text{curve}}\\ \;+\lambda_{8}L_{\text{unc}}+\lambda_{9}L_{\text{temp}} \end{array}$$ (29)

where the hyperparameter weights were empirically set to λ_1_ = 1.0, λ_2_ = 0.30, λ_3_ = 0.10, λ_4_ = 0.15, λ_5_ = 0.10, λ_6_ = 0.10, λ_7_ = 0.05, λ_8_ = 0.05, λ_9_ = 0.05. Each loss component was computed independently and combined using fixed weighting coefficients during optimization.

1. Coordinate regression loss: the lane coordinates were optimized using the smooth-L1 loss (Huber Loss) function (Girshick, 2015) and was calculated only for the lanes with valid annotations. The invalid lane coordinates were not optimized using the binary validity mask. The coordinate regression loss is expressed as in Equation 30:
$$\begin{array}{l} L_{coord}=\frac{1}{N}\underset{i}{\sum }M_{i}\cdot \text{SmoothL1}\left({\hat{x}}_{i},x_{i}\right) \end{array}$$ (30)
  where *M*_*i*_ denotes the validity mask, *x*_*i*_ represents the ground-truth coordinate, and ${\hat{x}}_{i}$ denotes the predicted coordinate.
2. Curvature and smoothness loss: to suppress abrupt coordinate variations and enforce natural curvature, a second-order finite difference was computed along each predicted lane, Equation 31:
$$\begin{array}{l} L_{smooth}=\frac{1}{N}\sum {\left({\hat{x}}_{i+1}-2{\hat{x}}_{i}+{\hat{x}}_{i-1}\right)}^{2}. \end{array}$$ (31)
  This regularization encouraged locally smooth lane trajectories and physically plausible lane shapes during optimization, acting as a strong geometric prior (Tabelini et al., 2021).
3. Confidence loss: confidence prediction branch was trained using binary cross-entropy loss. Confidence targets were acquired using validity mask of the lane annotations, which helped the model to predict the visibility of key-points of lanes simultaneously with coordinate prediction. The confidence loss is expressed as Equation 32.
$$\begin{array}{l} L_{conf}=\mathrm{BCE}\left(\hat{\text{C}},\text{C}\right), \end{array}$$ (32)
  where **C** denotes the binary confidence target derived from the ground-truth lane annotations.
4. Structural Topology Loss: To enforce the order of adjacent lanes, structural topology regularization was added. Lane center was defined by averaging horizontal coordinates of each predicted lane, and negative distance between neighboring lanes was penalized using rectified linear function, as in Equation 33:
$$\begin{array}{l} L_{top}=\text{ReLU}\left(-\left(c_{i+1}-c_{i}\right)\right) \end{array}$$ (33)
  where *c*_*i*_ denotes the center of the *i*^*th*^ lane. This constraint discourages lane crossings during optimization.
5. Lane separation and width loss: to encourage a consistent spatial arrangement between neighboring lanes, the lane centers were first computed by averaging the predicted horizontal coordinates across the predefined row anchors. The inter-lane spacing was then calculated between consecutive lane centers. The separation loss was defined as the mean squared deviation of the predicted spacing from its batch-level mean as in Equation 34 and the batch-level mean lane spacing is computed as Equation 35:
$$\begin{array}{l} L_{\text{sep}}=\frac{1}{B\left(L-1\right)}\overset{B}{\underset{b=1}{\sum }}\overset{L-1}{\underset{l=1}{\sum }}{\left(s_{b,l}-\bar{s}\right)}^{2}, \end{array}$$ (34)
  where
$$\begin{array}{l} c_{b,l}=\frac{1}{K}\overset{K}{\underset{k=1}{\sum }}{\hat{x}}_{b,l,k},\;s_{b,l}=c_{b,l+1}-c_{b,l}, \end{array}$$ (35)
  and $\bar{s}$ denotes the mean inter-lane spacing computed over the batch. The reference spacing is detached from the computational graph during optimization.
  The lane-width loss uses the same center-based formulation to penalize deviations from the mean neighboring-lane width, Equation 36:
$$\begin{array}{l} L_{\text{width}}=\frac{1}{B\left(L-1\right)}\overset{B}{\underset{b=1}{\sum }}\overset{L-1}{\underset{l=1}{\sum }}{\left(w_{b,l}-\bar{w}\right)}^{2}, \end{array}$$ (36)
  where *w*_*b, l*_ = *c*_*b, l*+1_−*c*_*b, l*_ represents the predicted width between neighboring lane centers and $\bar{w}$ denotes its batch-level mean. As implemented, $L_{\text{sep}}$ and $L_{\text{width}}$ use the same center-to-center spacing formulation, with separate loss weights in the total objective.
6. Curvature Loss: To constrain abrupt changes in the predicted lane trajectory, a second-order finite difference was computed along the row-anchor positions. The curvature loss is defined as in Equation 37:
$$\begin{array}{l} L_{\text{curve}}=\frac{1}{BL\left(K-2\right)}\overset{B}{\underset{b=1}{\sum }}\overset{L}{\underset{l=1}{\sum }}\overset{K-1}{\underset{k=2}{\sum }}{\left({\hat{x}}_{b,l,k+1}-2{\hat{x}}_{b,l,k}+{\hat{x}}_{b,l,k-1}\right)}^{2}, \end{array}$$ (37)
  where ${\hat{x}}_{b,l,k}$ denotes the predicted normalized horizontal coordinate of lane *l* at row anchor *k*. This second-order regularization encourages smooth variation of lane trajectories across consecutive row anchors.
7. Uncertainty-aware heteroscedastic loss: the uncertainty estimator predicts a positive variance value for each graph node to quantify the uncertainty associated with the corresponding lane keypoint representation. Rather than directly minimizing the predicted variance, which may encourage a degenerate low-variance solution, the proposed framework models uncertainty through an error-dependent heteroscedastic Gaussian negative log-likelihood (NLL). The uncertainty loss is defined as Equation 38:
$$\begin{array}{l} L_{unc}=\frac{1}{N_{v}}\underset{i\in V}{\sum }\frac{1}{2}\left[\frac{{\left({\hat{x}}_{i}-x_{i}\right)}^{2}}{v_{i}+\epsilon }+\log\left(v_{i}+\epsilon \right)\right], \end{array}$$ (38)
  where ${\hat{x}}_{i}$ and *x*_*i*_ denote the predicted and ground-truth normalized horizontal coordinates, respectively, *v*_*i*_ represents the predicted variance, V denotes the set of valid ground-truth keypoints, *N*_*v*_ is the number of valid keypoints, and ϵ is a small positive constant for numerical stability. The first term couples the predicted variance to the localization error, while the logarithmic term penalizes excessively small variance values when the associated localization error is non-zero, thereby discouraging a degenerate low-variance solution. Invalid lane points are excluded using the same validity mask used for coordinate regression. The resulting uncertainty estimates are subsequently used to derive confidence values through *C*_*i*_ = exp(−*v*_*i*_) for uncertainty-aware graph construction and feature weighting.
8. Temporal Graph Consistency Loss: A temporal regularization term was introduced to encourage consistency between graph representations of consecutive frames. Mean squared error was minimized between the graph representations, as expressed in Equation 39:
$$\begin{array}{l} L_{temp}=\text{MSE}\left(R_{t},R_{t-1}\right) \end{array}$$ (39)
  where *R*_*t*_ and *R*_*t*−1_ denote the confidence-weighted graph representations of the current and previous frames, respectively.
  The total loss was minimized using backpropagation during network training, allowing all network parameters to be optimized jointly.

## 4 Training procedure

### 4.1 Data preparation

The proposed architecture was trained through a supervised learning approach, where each training sample is formed by a current frame, two previous frames, and their respective lane annotations. All the input images were resized to have a fixed resolution of 800 × 288 pixels during data preprocessing. Then, pixel intensities were represented in RGB color space, converted to tensors, and standardized by means of ImageNet statistics (Deng et al., 2009) to feed the backbone network. In order to capture temporal information, a three-frame sequence was built for each training sample. The current frame was taken along with two previous frames. If some frames did not exist, then the available earlier frames were repeated to keep a fixed length of the temporal sequence. With such an approach, temporal transformer will receive sequences with identical shape independent of frame position. Lane annotations were represented using a row-anchor formulation (Qin et al., 2020). For the purpose of representing lane annotations, a row anchor approach was used. Twenty-five fixed row-anchors were uniformly distributed between 40% and 100% of the image height following the row-anchor formulation. The same anchor positions were used for every training and testing image. In order to obtain lane coordinates, points belonging to every annotated lane were sorted based on their vertical coordinates and linear interpolation was carried out to get the horizontal coordinate of the lane at every defined row anchor. In case a lane did not have any points at certain row height, coordinate value of −1 was assigned to it, denoting the absence of any lane point there.

### 4.2 Network initialization

Initialization of this framework was done by employing randomly generated learnable node embeddings and row embeddings that were optimized together throughout the process of training. Feature extraction of the spatial features was done using a light-weight convolutional backbone consisting of convolution, batch normalization, ReLU activation, and residual blocks (Huang et al., 2017). Subsequently, the obtained feature maps were mapped to the graph feature space through the use of the global average pooling followed by linear projection. Training of the network parameters was carried out using the AdamW optimizer (Loshchilov and Hutter, 2017), where the learning rate was set to be 3 × 10^−4^ with a weight decay of 1 × 10^−4^. The batch size of four was used for all experiments. At most 50 training epochs were used in training, but the training can end before reaching the maximum number of epochs.

### 4.3 Loss optimization

The proposed architecture was tuned based on the composite loss function described in Section 3.9. For every epoch of training, coordinate regression loss, confidence loss, geometric regularization, uncertainty-aware heteroscedastic loss, and graph temporal consistency loss were evaluated and aggregated with pre-defined weights. This aggregate loss was then propagated back through the network to tune all the learnable parameters.

### 4.4 Model optimization

Updates on the network parameters were achieved via the standard backpropagation process involving the composite loss function. Following the calculation of the gradients, the gradients were clipped with a maximum norm of 1.0 in order to stabilize the optimization process and mitigate exploding gradients while learning graph attention. Adaptive adjustment on the learning rate was done via the ReduceLROnPlateau scheduling. The learning rate was reduced by a factor of 0.5 whenever the validation loss could not be improved over one validation cycle. Early stopping was performed in order to prevent the overfitting problem. Training was stopped if the validation loss did not improve over seven consecutive epochs. Besides, the checkpointing was done to save the trained model after each epoch where the best-performing model with minimum validation loss was automatically saved. The complete training procedure adopted for optimizing the model is summarized in Algorithm 2. The algorithm outlines the optimization process performed during each training epoch. The optimized model corresponding to the lowest validation loss was retained and subsequently used for inference and performance evaluation.

Algorithm 2 Model training procedure. 
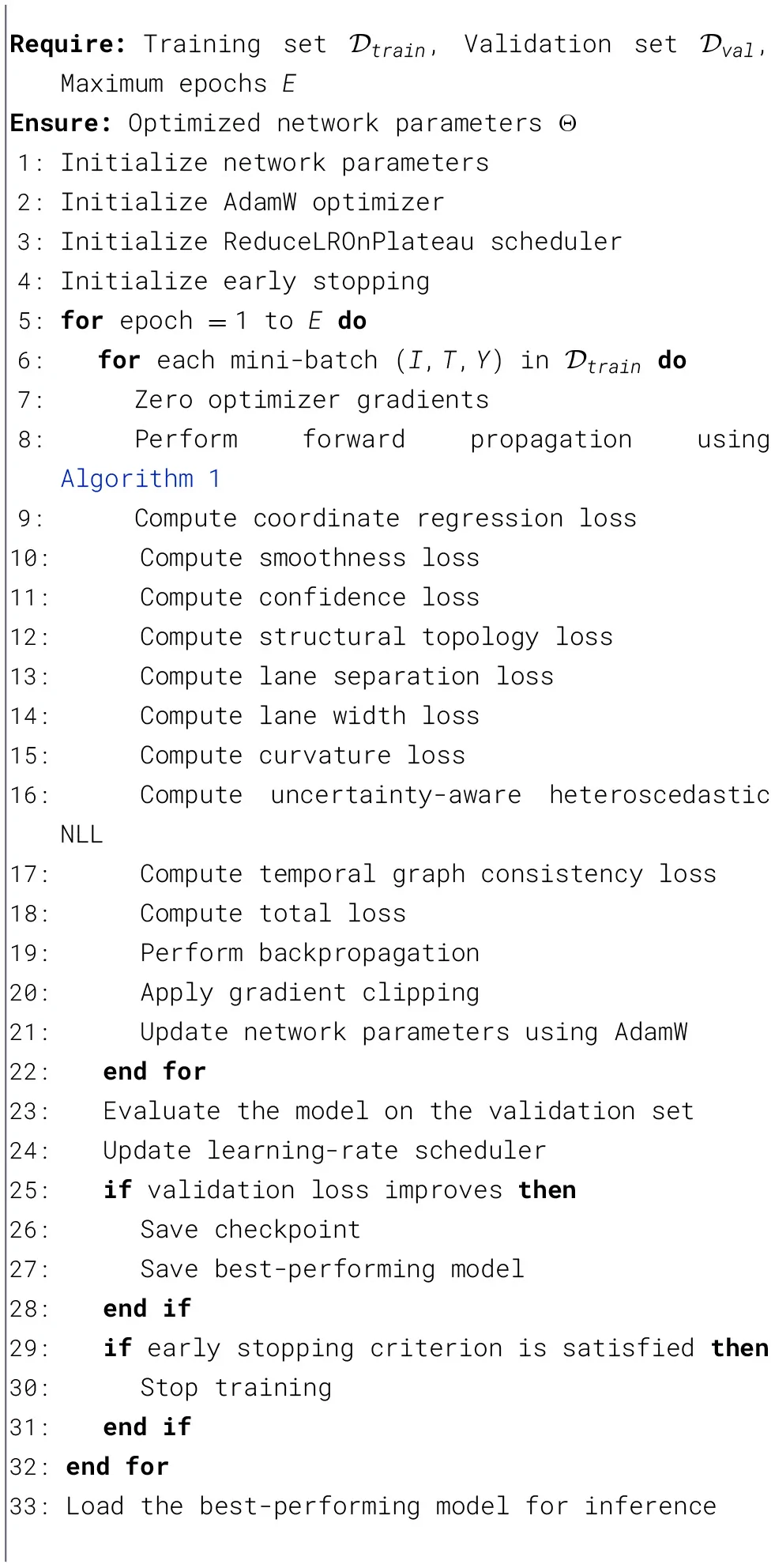

### 4.5 Inference

In inference time, only the forward pass of the proposed architecture was executed. The current frame, together with the sequence corresponding to it, was fed to the network in order to make the predictions regarding the lanes' coordinates and their confidence. Lane's coordinates prediction was made by the coordinate regression part while the confidence part of the network gave the prediction of the confidence of visibility of each lane point.

## 5 Experimental setup

### 5.1 Experimental environment

All the experiments were performed via the Kaggle cloud-based computation infrastructure that is powered by an NVIDIA GPU Tesla P100. The developed framework was implemented in PyTorch, whereas OpenCV was used for image processing and annotations. CUDA was used to utilize GPU acceleration, and all experiments were carried out via the Python programming language. Computational measurements were performed on the Tesla P100 using a batch size of one.

### 5.2 Datasets

The proposed framework was trained and evaluated using the CULane dataset (Pan et al., 2018), which provides diverse urban driving scenes containing normal roads, crowded traffic, shadows, night conditions, intersections, and lane occlusions. The official training, validation, and testing splits were preserved throughout all experiments. The dataset was accessed through a Kaggle mirror for convenient download while preserving the official training, validation, and testing splits provided by the original CULane benchmark.

### 5.3 Implementation details

The implementation settings used in all experiments are listed in Table 2. These hyperparameters were fixed in all training and testing experiments except when otherwise specified. The hyperparameters were chosen empirically from preliminary experiments, whereas the effect of main hyperparameters was analyzed in sensitivity analysis (Section 6.9). The network model accepted images of size 800 × 288 pixels and operated on three successive frames. A total of four lanes can be extracted in an image, where each lane had 25 row-anchor keypoints. The temporal information was modeled using a transformer network with two encoder layers and eight attention heads. Graph reasoning was performed using two layers of graph attention networks with four attention heads. In dynamic graph construction, Top-*k* pruning ensured that eight nodes were considered as neighbors of each node.

**Table 2:** Implementation Hyperparameters of the Proposed GraphLane.

Hyperparameter	Value
Input image resolution	800 × 288
Temporal frames	3
Number of lanes	4
row-anchors	25
Graph hidden dimension	128
Graph attention layers	2
Graph attention heads	4
Transformer layers	2
Transformer attention heads	8
Top-*k* neighbors	8
Optimizer	AdamW
Learning rate	3 × 10^−4^
Weight decay	1 × 10^−4^
Batch size	4
Maximum epochs	50
Gradient clipping	1.0
Learning-rate scheduler	ReduceLROnPlateau
Scheduler patience	1
Scheduler factor	0.5
Early stopping patience	7

### 5.4 Evaluation metrics

The proposed approach was evaluated using lane-existence and coordinate-based metrics, including Precision, Recall, F1-score, Accuracy, Intersection over Union (IoU), Mean Absolute Error (MAE), Root Mean Squared Error (RMSE), and Average Localization Error (ALE). Precision, Recall, F1-score, Accuracy, and IoU were used to evaluate lane-existence prediction, whereas MAE, RMSE, and ALE were used to quantify the accuracy of the predicted lane coordinates. The F1-score reported in this table corresponds to the lane-existence evaluation defined in Section 5.4 for the proposed row-anchor-based representation. It should not be interpreted as the official CULane benchmark F1-score or as a state-of-the-art result on CULane.

#### 5.4.1 Evaluation protocol

The proposed framework predicts four lane instances, with each lane represented by 25 keypoints sampled at uniformly spaced vertical row anchors between 0.40 and 1.00 of the image height. During dataset preparation, the ground-truth lane coordinates are normalized by the original image width. If a ground-truth lane annotation does not span a particular row anchor, the corresponding ground-truth coordinate is assigned a value of −1 and treated as invalid. Such invalid ground-truth keypoints are excluded from coordinate-based error calculations. The 25 row anchors and the handling of unavailable annotation points are applied consistently to all evaluated samples.

For lane-existence evaluation, the predicted lane slots are compared with the corresponding ground-truth lane slots according to their fixed lane indices. Specifically, predicted lane *l* is compared with ground-truth lane *l*, where the lane ordering follows the lane ordering in the ground-truth annotation. No additional geometric lane-matching, nearest-neighbor assignment, or point-to-point distance threshold is applied. A predicted lane is considered present when its mean confidence across the 25 keypoints exceeds 0.4, as defined in Equation 40:


$$\begin{array}{l} E_{l}^{pred}=⊮\left(\frac{1}{25}\overset{25}{\underset{k=1}{\sum }}c_{l,k}>0.4\right), \end{array}$$ (40)

where *c*_*l, k*_ denotes the predicted confidence of the *k*-th keypoint of lane *l*. A ground-truth lane is considered present when at least one valid ground-truth point is available, as defined in Equation 41:


$$\begin{array}{l} E_{l}^{gt}=⊮\left(\overset{25}{\underset{k=1}{\sum }}⊮\left(g_{l,k}\geq 0\right)>0\right). \end{array}$$ (41)

Thus, the only threshold used for lane-existence classification is a confidence threshold of 0.4. No geometric distance threshold is used for lane-slot correspondence. True positives (TP), false positives (FP), false negatives (FN), and true negatives (TN) are obtained by comparing the predicted and ground-truth lane-existence decisions. A lane with invalid keypoints in the ground truth is not automatically considered a false detection; its lane-existence status is determined by whether at least one valid ground-truth keypoint is available.

Precision, Recall, and F1-score are calculated as Equations 42–44


$$\begin{array}{l} Precision=\frac{TP}{TP+FP}, \end{array}$$ (42)

$$\begin{array}{l} Recall=\frac{TP}{TP+FN}, \end{array}$$ (43)

and


$$\begin{array}{l} F1=\frac{2\left(Precision\right)\left(Recall\right)}{Precision+Recall}. \end{array}$$ (44)

Accuracy is calculated as Equation 45


$$\begin{array}{l} Accuracy=\frac{TP+TN}{TP+TN+FP+FN}. \end{array}$$ (45)

The IoU reported in this work is calculated from the lane-existence decisions as Equation 46


$$\begin{array}{l} IoU=\frac{TP}{TP+FP+FN}. \end{array}$$ (46)

Accordingly, this measure represents the Jaccard overlap between the predicted and ground-truth lane-existence sets rather than pixel-level or geometric overlap between lane curves. It is therefore referred to as lane-existence IoU in the evaluation protocol.

For coordinate-based localization evaluation, only valid ground-truth keypoints are considered. Let


$$M=\left\{\left(l,k\right):g_{l,k}\geq 0\right\}$$

denote the set of valid ground-truth lane keypoints. The normalized mean absolute error (MAE) is calculated as Equation 47


$$\begin{array}{l} MAE=\frac{1}{\left|M\right|}\underset{\left(l,k\right)\in M}{\sum }|{\hat{x}}_{l,k}-x_{l,k}|, \end{array}$$ (47)

where ${\hat{x}}_{l,k}$ and *x*_*l, k*_ denote the predicted and ground-truth normalized *x*-coordinates, respectively. The Root Mean Squared Error (RMSE) is calculated over the same set of valid keypoints as Equation 48


$$\begin{array}{l} RMSE=\sqrt{\frac{1}{\left|M\right|}\underset{\left(l,k\right)\in M}{\sum }{\left({\hat{x}}_{l,k}-x_{l,k}\right)}^{2}}. \end{array}$$ (48)

Therefore, ground-truth coordinates represented by −1 are excluded from MAE and RMSE calculations, and only valid annotated keypoints contribute to the localization errors. Because the predicted coordinates are normalized by the original image width, the normalized MAE can be expressed in the original image coordinate system by multiplying it by the image width of 800 pixels. The corresponding pixel-domain localization error is therefore expressed as Equation 49


$$\begin{array}{l} ALE_{px}=MAE\times 800. \end{array}$$ (49)

Thus, ALE is the pixel-domain representation of the normalized MAE and is reported to provide an interpretable localization error in pixels rather than as an independent error formulation.

### 5.5 Graph adaptation analysis

To quantify the adaptivity of the Dynamic Temporal Graph Construction (DTGC) module, the selected edge sets were analyzed across the official CULane test-condition subsets. For each input sample, self-loops were excluded and the upper-triangular portion of the symmetric adjacency matrix was used to represent the undirected edge set. The Jaccard similarity between consecutive edge sets was calculated as the ratio of their intersection to their union. The corresponding edge-change rate was defined as 1−*J*, where *J* denotes the Jaccard similarity. Higher edge-change values therefore indicate greater variation in the selected graph connections between consecutive samples. The analysis was performed separately for the Normal, Crowd, Hlight, Shadow, Noline, Arrow, Curve, Cross, and Night conditions using the official CULane test-condition lists.

## 6 Results and discussion

In this part, the performance of the framework is tested quantitatively and qualitatively using thorough experiments carried out on the CULane benchmark dataset. Special attention is given to the accuracy of the localization of lanes' coordinates, which is crucial for the correct functioning of autonomous driving. First, the proposed framework is tested quantitatively through localization and lane detection metrics, followed by a qualitative test on different driving cases. Then, the performance of the framework is compared with the baselines such as CNN Baseline, Fixed GAT, Temporal, and GCN. After that, an ablation study is provided to show the importance of every proposed component. Computational complexity is finally analyzed.

### 6.1 Quantitative performance of the proposed framework

The framework was evaluated on the CULane test set using the row-anchor-based evaluation protocol described in Section 5.4. This evaluation is used to assess the proposed lane representation and is distinct from the official CULane benchmark evaluation protocol. The model achieving the lowest validation loss during training was selected for testing. Since the model is primarily designed to improve lane coordinate localization while maintaining reliable lane detection, both localization and detection performances were evaluated. The overall quantitative results are summarized in Table 3. The F1-score reported in this table corresponds to the lane-existence evaluation defined in Section 5.4 for the proposed row-anchor-based representation. It should not be interpreted as the official CULane benchmark F1-score or as a state-of-the-art result on CULane.

**Table 3:** Overall performance of the proposed framework on the CULane test set.

Metric	Value
Precision	0.9769
Recall	0.8990
F1-score	0.9363
Accuracy	0.9072
Lane-existence IoU	0.8803
MAE	0.0241
RMSE	0.0464
Average localization error (pixels)	19.28

Firstly, the localization accuracy of the proposed framework was examined using MAE, RMSE, and average localization error (pixels) criteria. In this case, the model reached an MAE of 0.0241, an RMSE of 0.0464, and an Average Localization Error of 19.28 pixels. Such results suggest that the estimated lane coordinates are very close to the actual coordinates of annotated lanes with minor localization errors. Therefore, the low localization error indicates the success of the integration of temporal context, confidence-weighted graph reasoning, and geometry-aware optimization. Along with high localization accuracy, the proposed framework also showed a good lane detection performance with the adopted evaluation protocol. Specifically, the framework reached a precision of 0.9769, a recall of 0.8990, and an F1-score of 0.9363, having achieved an accuracy of 0.9072 and an IoU of 0.8803. Consequently, these results prove that the framework can both reliably estimate lane geometry and predict lane existence at the same time. Overall, the quantitative evaluation demonstrates that the proposed framework effectively balances lane localization accuracy and lane detection performance. The obtained results indicate that the proposed uncertainty-guided spatio-temporal graph learning framework can accurately estimate lane coordinates while preserving robust lane detection capability across diverse driving scenes.

To further characterize the behavior of GraphLane under different driving conditions, an additional condition-wise evaluation was conducted on the complete official CULane test split containing 34,680 images. The same row-anchor-based evaluation formulation described in Section 5.4 was retained to ensure consistency with the principal experiments. The results are summarized in Table 4. The condition-wise results show that GraphLane maintains consistent lane-existence performance across most of the evaluated driving conditions under the proposed representation. The highest lane-existence F1-score was obtained for the Normal condition (0.9527), followed by Arrow (0.9435), Hlight (0.9406), Crowd (0.9238), Shadow (0.9184), and Curve (0.9124) conditions. Performance was comparatively lower for Noline (0.8803) and Night (0.8607), which involve greater visual ambiguity and reduced lane visibility. The corresponding Average Localization Error values were 30.28 pixels for Normal scenes and ranged from 34.40 to 55.03 pixels across the remaining conditions with valid ground-truth lane annotations. These results indicate that the proposed row-anchor representation provides stable lane-existence and localization performance across diverse scene conditions, while the degradation observed under Noline and Night conditions highlights the remaining difficulty of lane localization when lane markings are weak or visibility is reduced.

**Table 4:** Condition-wise evaluation of GraphLane on the official 34,680-image CULane test split using the proposed row-anchor-based evaluation.

Condition	Images	Precision	Recall	F1	Accuracy	IoU	MAE	RMSE	ALE (px)
Normal	9,621	0.9877	0.9201	0.9527	0.9222	0.9097	0.0379	0.0619	30.28
Crowd	8,113	0.9835	0.8710	0.9238	0.8760	0.8584	0.0538	0.0868	43.05
Hlight	486	0.9832	0.9015	0.9406	0.9012	0.8878	0.0486	0.0787	38.86
Shadow	930	0.9669	0.8745	0.9184	0.8798	0.8491	0.0528	0.0760	42.27
Noline	4,067	0.9792	0.7996	0.8803	0.8126	0.7862	0.0686	0.1014	54.90
Arrow	890	0.9951	0.8969	0.9435	0.9039	0.8930	0.0430	0.0698	34.40
Curve	422	0.9651	0.8651	0.9124	0.8709	0.8389	0.0688	0.1019	55.03
Cross	3,122	–	–	–	0.7500	–	–	–	–
Night	7,029	0.9193	0.8092	0.8607	0.8041	0.7555	0.0659	0.0930	52.68
Overall	34,680	0.9399	0.8657	0.9013	0.8566	0.8203	0.0529	0.0827	42.34

F1, Precision, Recall, Accuracy, and IoU are based on lane-existence decisions for the four fixed lane slots, while MAE, RMSE, and ALE quantify coordinate localization under the proposed 25-row-anchor representation. The Cross subset contains 3,122 images but no valid positive ground-truth lane instances under this representation (0 valid GT images and 0 valid row-anchor points). Therefore, Precision, Recall, F1, IoU, and localization-error metrics are not applicable and are reported as “–”. The reported values are internal row-anchor-based evaluation results and are not official CULane benchmark scores.

The Cross condition requires separate interpretation. Although it contains 3,122 test images, none contains a valid positive lane instance under the fixed four-lane, 25-row-anchor representation used in this study. Consequently, positive-class metrics such as precision, recall, F1-score, IoU, and coordinate localization errors are not applicable for this subset and are reported as “–”. The 75.00% accuracy corresponds only to the four-slot lane-existence classification and should therefore not be interpreted as a lane-detection F1 result. The overall result on the complete official test split was a lane-existence F1-score of 0.9013 with an Average Localization Error of 42.34 pixels.

### 6.2 Statistical reliability analysis

The stability of the suggested framework was assessed by carrying out bootstrap resampling on the CULane test set using 1,000 bootstrap runs. In this way, the 95% confidence intervals were estimated for the key metrics used for evaluation, namely Precision, Recall, F1-score, and Average Localization Error (pixels). Table 5 reports the bootstrap mean, standard deviation, and 95% confidence interval of each evaluation metric across the resampled test sets.

As shown in Table 5, the proposed GraphLane framework exhibits stable performance across the 1,000 bootstrap resampling iterations. The F1-score achieves a bootstrap mean of 0.9363 with a narrow 95% confidence interval of [0.9339, 0.9387], indicating consistent lane detection performance across the resampled test sets. Similarly, the Average Localization Error (pixels) records a bootstrap mean of 19.27 pixels with a 95% confidence interval of [18.80, 19.74] pixels. The relatively small standard deviations and narrow confidence intervals indicate that the proposed framework maintains reliable and statistically stable performance across different bootstrap samples of the CULane test set. The bootstrap means differ slightly from the corresponding point estimates reported in the main experimental results because they represent the average performance across 1,000 resampled test sets rather than the metrics computed on the original test set.

**Table 5:** Bootstrap statistical reliability analysis on the CULane test set (1,000 bootstrap iterations).

Metric	Mean	Std	95% Confidence interval
Precision	0.9768	0.0013	[0.9743, 0.9796]
Recall	0.8991	0.0019	[0.8953, 0.9029]
F1-score	0.9363	0.0012	[0.9339, 0.9387]
Average localization error (pixels)	19.27	0.24	[18.80, 19.74]

### 6.3 Qualitative analysis

In order to assess the practicality of the proposed framework, qualitative tests were performed using selected images of the CULane test dataset. Figures 5, 6 illustrate lane detection outcomes for various driving situations, including city driving, busy scenes, partially occluded lanes, presence of shadows, significant illumination changes, night-time driving, and scenes where there is no lane marking. In each sample image, both the original image, the corresponding ground truth, and predicted lane coordinates are shown.

**Figure 5:**
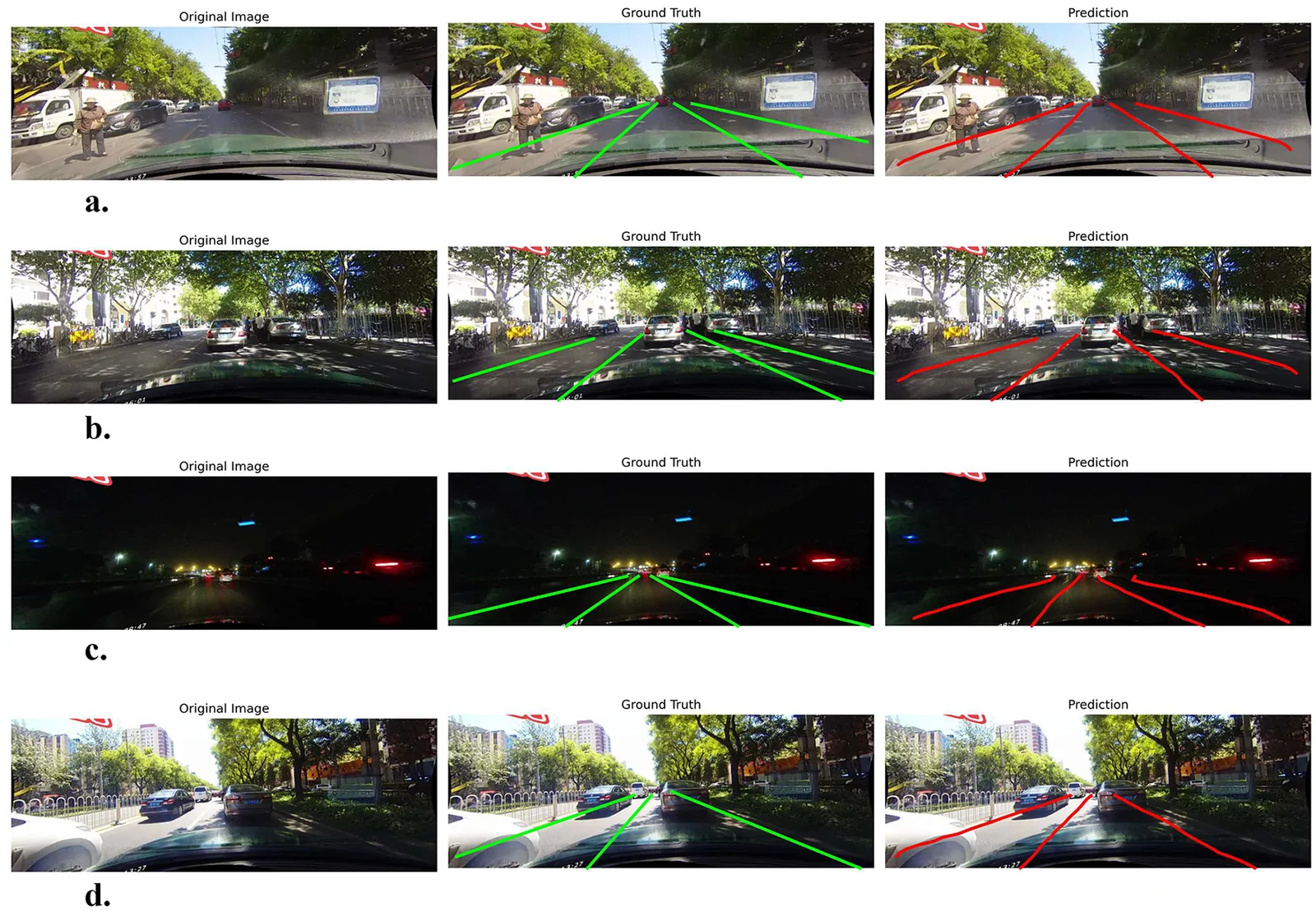
Representative qualitative lane localization results of the proposed model on the CULane test set. From left to right: original image, ground-truth lane annotations, and predicted lane coordinates. **(a)** Urban road scene. **(b)** Tree-shadowed roadway. **(c)** Low-light highway. **(d)** Strong illumination.

**Figure 6:**
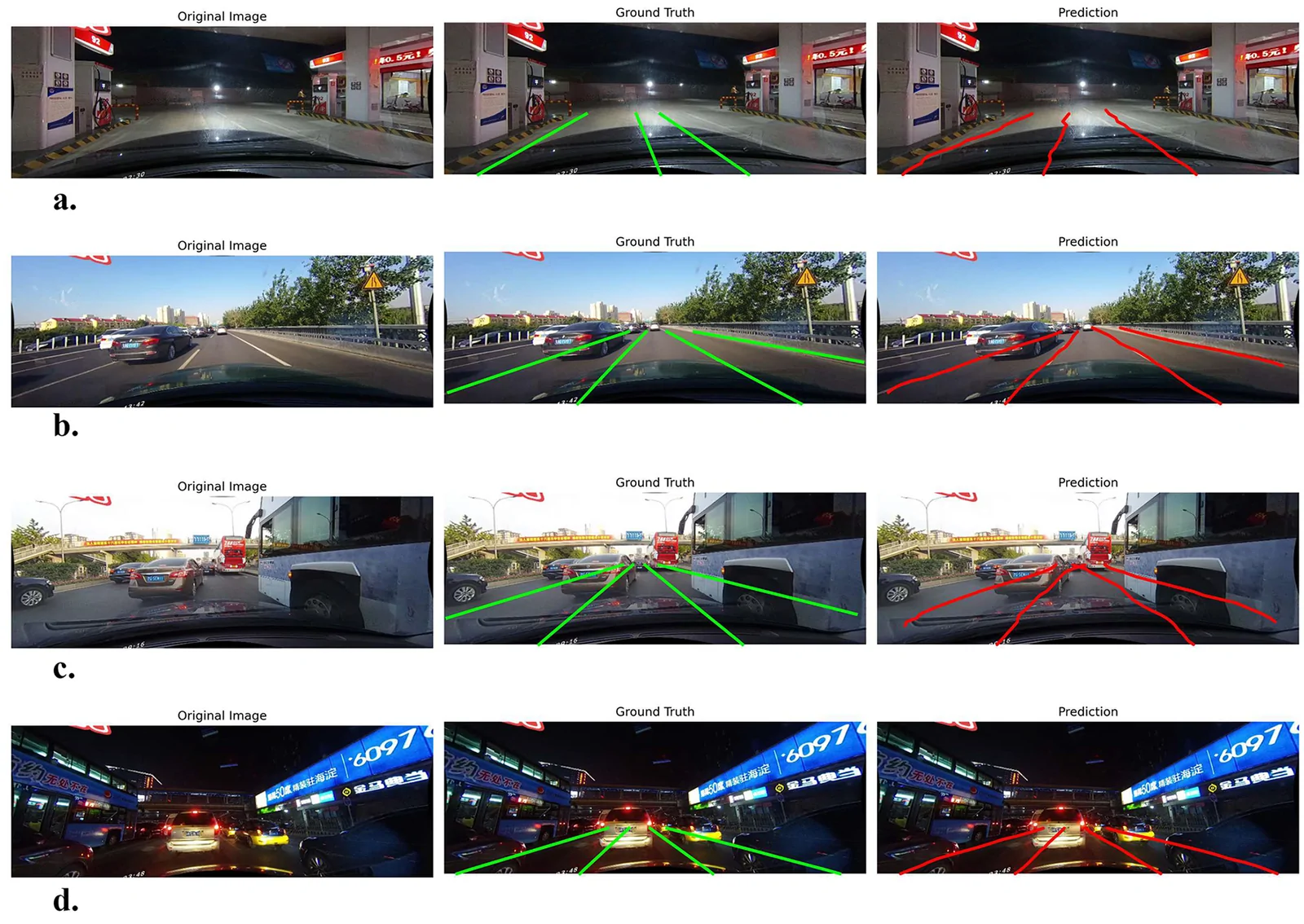
Qualitative visualization of lane localization by proposed model on representative CULane test images. **(a)** Nighttime urban driving. **(b)** Daytime highway driving. **(c)** Congested traffic. **(d)** Low-light urban scene.

As seen from Figures 5, 6, the proposed model consistently predicts lanes with coordinates closely following the annotated geometry under different driving conditions. Moreover, the coordinates of the predicted lanes remain smooth and connected even when several cars are present in the scene, when there are partially occluded lanes, and in the case of urban driving. It indicates that the temporal feature aggregation contributes to maintaining lane continuity in the illustrated sequences using the information from successive video frames, while the graph reasoning model identifies structural relationships between lanes. The designed approach also shows good robustness in adverse illumination scenarios. With a scenario involving heavy tree shadows, bright daylight, and night scenes, the predicted lane coordinates still match the ground-truth annotations accurately despite low visibility of the lane markings. The uncertainty-driven dynamic graph generation reduces the contribution of interactions associated with high-uncertainty nodes, allowing the graph attention layers to place greater emphasis on more reliable node relationships. This ensures that the localization of lanes continues to be accurate even though the lane markings are partially damaged or inconsistent. Additionally, the designed approach shows stable performance in traffic congestion scenarios whereby the nearby vehicles partially obscure the lane markings. Unlike many approaches that provide fragmented lane detection results, the approach provides continuous geometry of lanes by combining spatio-temporal information with the spatial graph reasoning. Another qualitative example is given for the case of a road scene without annotated lanes. For the current case, the proposed framework successfully avoids making any lane predictions, which supports that the confidence estimation module is able to avoid detecting any false positive detections in case there are no lane markings on the road scene. Thus, the framework not only learns how to estimate the correct coordinates of the lanes but also to understand when lane predictions have to be avoided. Overall, the qualitative results demonstrate that the proposed uncertainty-guided spatio-temporal graph learning framework achieves robust and geometrically consistent lane localization across diverse real-world driving environments. The visual agreement between the predicted lane coordinates and the ground-truth annotations further supports the quantitative results presented in the previous subsection.

### 6.4 Dynamic graph behavior analysis

In order to understand the internal mechanism of decision making in GraphLane, the behavior of the Dynamic Temporal Graph Construction (DTGC) module is studied based on the entire CULane test set. Representative qualitative examples are illustrated in Figure 7, while quantitative statistics over the entire CULane test set are summarized in Table 6. As shown in Table 6, the learned graph has a mean graph density of 0.1567 ± 0.0033, indicating that only approximately 15.7% of the possible node connections remain active after Top-k sparsification. This demonstrates that the DTGC module produces sparse graph representations while retaining selected relationships among lane-keypoint nodes. Since the Top-*k* value is fixed, the graph density itself is constrained by the sparsification setting and therefore does not independently establish graph adaptivity. The mean prediction confidence of 0.5022 ± 0.1890 and mean predicted uncertainty of 0.008078 ± 0.015045 indicate that the model produces non-uniform reliability estimates across lane-keypoint predictions. These estimates are incorporated into confidence-weighted graph construction according to Equations 15–18, where learned edge strengths are first obtained through the Edge MLP and sigmoid activation, subsequently modulated by node confidence, sparsified using Top-k selection, and symmetrized before graph reasoning. The mean temporal graph difference of 0.006000 ± 0.006388 indicates that the selected graph topology generally changes only slightly between consecutive inputs, while the maximum temporal graph difference of 0.4823 ± 0.2395 shows that substantially larger topology changes can occur for individual samples. This combination suggests that DTGC maintains relative temporal stability while retaining the ability to modify graph connectivity when the input scene changes. The Edge-MLP output magnitude further indicates that the dynamic graph module learns differentiated pairwise relationships before sigmoid normalization. Overall, the results support the ability of DTGC to construct sparse and sample-dependent graph representations while confidence weighting suppresses unreliable node interactions and Top-k sparsification controls graph complexity.

**Figure 7:**
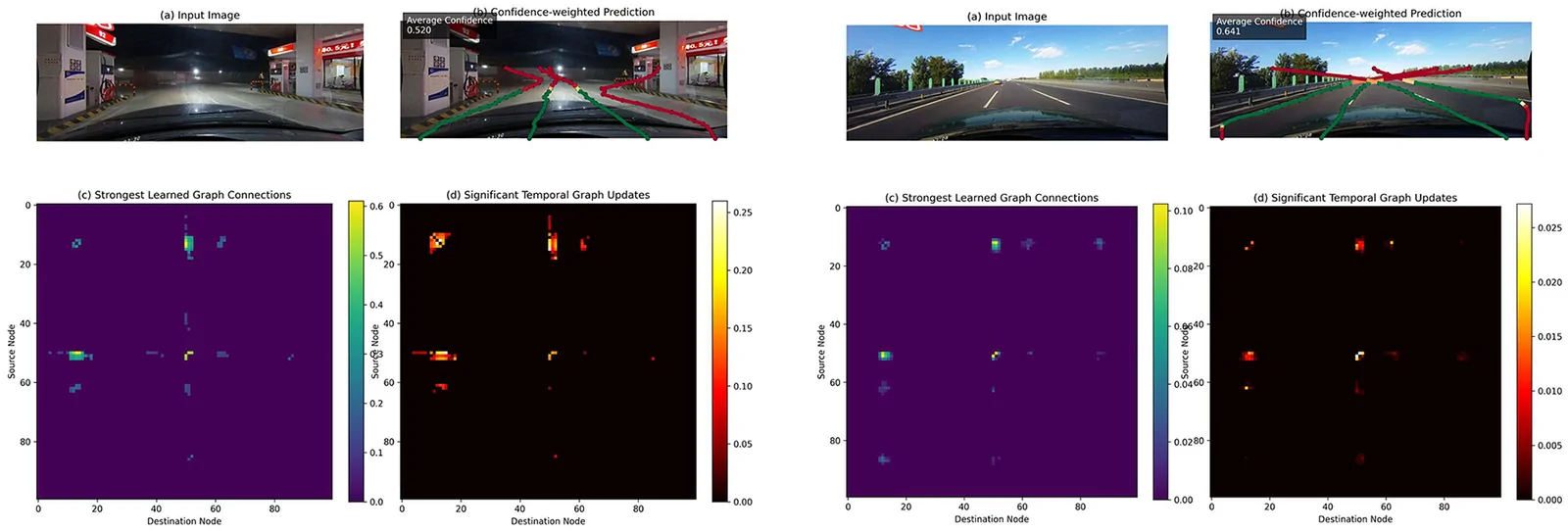
Representative visualization of the proposed GraphLane under different driving conditions. Each example shows **(a)** the input image, **(b)** confidence-weighted lane predictions, **(c)** the strongest learned graph connections after Top-*k* sparsification, and **(d)** temporal graph updates between consecutive frames. The visualizations demonstrate that the proposed Dynamic Temporal Graph Construction (DTGC) produces sparse graph structures while allowing the learned connectivity to vary across input scenes.

**Table 6:** Analysis of the dynamic graph behavior of the proposed GraphLane on the CULane test set.

Metric	Value	Interpretation
Mean prediction confidence	0.5022 ± 0.1890	The confidence estimation module produces varying confidence levels across lane-keypoint predictions, reflecting differences in prediction reliability.
Mean prediction uncertainty	0.008078 ± 0.015045	The uncertainty-aware formulation produces positive and non-uniform variance estimates across the evaluated lane-keypoint predictions.
Graph density	0.1567 ± 0.0033	Approximately 15.7% of possible node connections remain active after Top-*k* sparsification, resulting in sparse graph representations.
Temporal graph difference (mean)	0.006000 ± 0.006388	The graph topology exhibits limited average variation across consecutive inputs while allowing sample-dependent structural adaptation.
Temporal graph difference (maximum)	0.4823 ± 0.2395	The larger maximum difference indicates that the learned graph can undergo substantial topology changes for samples requiring greater structural adaptation.
Maximum Edge-MLP Logit	1.7161 ± 0.1710	The edge MLP produces differentiated pairwise relationship scores before sigmoid normalization.

The sparse graph representation indicates that the DTGC module retains a limited subset of the possible node relationships rather than constructing a dense graph. The mean graph density of 0.1567 ± 0.0033 shows that approximately 15.7% of possible connections remain active after Top-*k* sparsification. Since the sparsity level is constrained by the fixed Top-*k* setting, the graph density alone does not establish adaptivity; rather, the adaptive behavior is reflected in the learned edge identities and their variation across input samples. The mean temporal graph difference of 0.006000 ± 0.006388 indicates that the learned graph topology generally exhibits limited variation across consecutive inputs while retaining the ability to modify its connectivity when required. At the same time, the maximum temporal graph difference of 0.4823 ± 0.2395 demonstrates that substantially larger graph changes can occur for individual samples. This combination of relatively small average variation and larger sample-level changes suggests that the DTGC module maintains graph stability while allowing topology to adapt to changes in the input scene. The mean prediction confidence of 0.5022 ± 0.1890 further indicates that the model does not assign a uniform confidence value across lane-keypoint predictions. In conjunction with the mean predicted uncertainty of 0.008078 ± 0.015045, these results demonstrate that the uncertainty-aware mechanism produces non-uniform estimates that are subsequently incorporated into confidence-weighted graph construction. The maximum edge-MLP logit of 1.7161 ± 0.1710 further indicates the presence of stronger learned relationships among selected node pairs.

Overall, these results support the ability of the proposed DTGC module to construct sparse and sample-dependent graph representations. The graph remains constrained in size by Top-*k* sparsification, while the learned edge relationships can vary between input samples. The temporal graph statistics therefore provide quantitative evidence that GraphLane maintains a degree of structural stability while retaining the flexibility to modify graph connectivity when required. The qualitative examples in Figure 7 provide additional visual evidence of the learned sparse graph connections and their temporal variation across representative driving scenes.

### 6.5 Uncertainty analysis

To further assess whether the predicted uncertainty provides meaningful information about localization reliability, an uncertainty–error correlation analysis was performed on the validation set using the best-performing checkpoint. The analysis considered all valid lane keypoints and compared the predicted variance with the corresponding absolute localization error. A total of 490,041 valid keypoints were evaluated. The predicted variance exhibited a positive Pearson correlation of *r* = 0.5446 and a positive Spearman rank correlation of ρ = 0.6363, with *p* < 0.001 for both measures. These positive correlations indicate that keypoints assigned higher predicted uncertainty generally exhibit larger localization errors. Therefore, the learned uncertainty estimates provide informative reliability signals rather than collapsing to uniformly low variance values. The corresponding relationship is illustrated in Figure 8.

**Figure 8:**
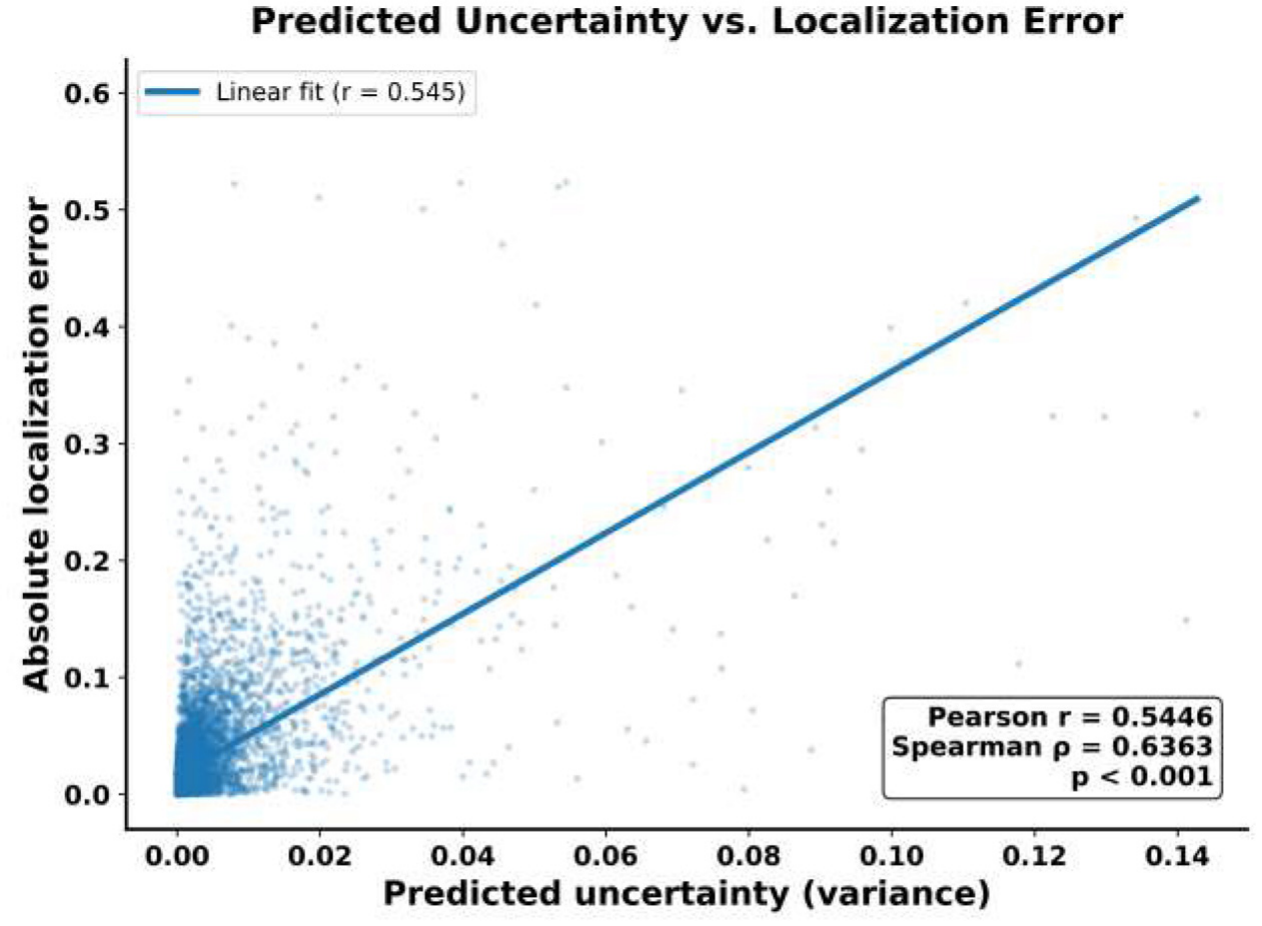
Relationship between predicted uncertainty and absolute localization error on the validation set. The visualization shows 10,000 randomly sampled valid keypoints, while the reported Pearson and Spearman correlations are computed using all 490,041 valid keypoints. The positive correlations indicate that higher predicted uncertainty is generally associated with larger localization errors.

### 6.6 Computational analysis

The computational complexity of the presented framework has been analyzed to determine the efficiency and memory of the inference process. The complexity of the model has been analyzed by evaluating the number of trainable parameters, checkpoint size, FLOPs, inference speed, and inference latency of the model using the trained network in Table 7. The computational complexity of the proposed framework was evaluated in terms of trainable parameters, checkpoint storage size, FLOPs, inference speed, and inference latency. The proposed framework contains 3.81 million trainable parameters. The checkpoint size of 43.73 MB corresponds to the size of the saved checkpoint file and is therefore reported separately from the parameter count. The computational cost is 26.34 GFLOPs for an input resolution of 800 × 288 pixels with three sequential input frames. Thus, the parameter count should not be interpreted as a direct conversion to the reported checkpoint size.

**Table 7:** Computational complexity of the proposed framework.

Metric	Value
Trainable parameters	3.81 M
Checkpoint storage	43.73 MB
FLOPs	26.34 GFLOPs
Inference speed	88.64 FPS
Inference latency	11.28 ms

For inference-speed measurement, a batch size of one was used. The three-frame temporal input was supplied to the model during each forward pass. The model was first warmed up for 50 forward passes, followed by 200 timed forward passes. The reported FPS and latency correspond only to the model forward-pass computation and exclude data loading, image preprocessing, and post-processing. Under this measurement protocol, the proposed framework achieved a mean inference speed of 88.64 FPS and a mean inference latency of 11.28 ms per forward pass. These measurements indicate that the proposed framework can process the three-frame input sequence with an inference latency of 11.28 ms per forward pass under the specified GPU evaluation setup.

### 6.7 Comparative evaluation

To provide a controlled evaluation of the proposed framework, four internal baseline architectures were implemented and trained under identical experimental conditions. Direct numerical comparison with published CULane results is not treated as a strictly equivalent benchmark comparison because published methods differ in network architecture, input resolution, training strategy, lane representation, and evaluation protocol. In particular, GraphLane represents each lane using four lane slots with 25 fixed row-anchor keypoints and evaluates lane existence from keypoint-level confidence, whereas published CULane studies may report benchmark scores using their own lane representations and evaluation procedures. Therefore, published results are not directly combined with the controlled results in Table 8. Instead, the internal baselines isolate the contribution of temporal modeling, graph reasoning, and dynamic topology under the same implementation and evaluation protocol.

**Table 8:** Quantitative comparison of localization accuracy and computational complexity.

	Localization metrics				Computational complexity			
Model	F1	Lane-existence IoU	MAE	RMSE	Average localization error (pixels)	Params (M)	FPS	Lat (ms)
CNN baseline	0.9308	0.8705	0.0372	0.0592	29.73	2.19	500.39	2.00
Temporal	0.9356	0.8791	0.0396	0.0624	31.71	3.77	163.25	6.13
GCN	0.8764	0.7800	0.0763	0.1047	61.05	3.63	158.01	6.33
Fixed GAT	0.9386	0.8897	0.0389	0.0607	31.10	3.73	153.09	6.53
**Proposed**	**0.9363**	**0.8803**	**0.0241**	**0.0464**	**19.28**	3.81	88.64	11.28

Bold values indicate the proposed/full framework configuration or the selected default parameter setting.

All models utilized an identical 800 × 288 input resolution and were trained using the same common coordinate, smoothness, and confidence loss components. Geometry-aware optimization was enabled only in the corresponding ablation configurations, as described in Section 6.8. Architecture-specific uncertainty and graph-temporal consistency terms were not applied to baseline models that did not explicitly model these quantities. The baseline architectures are defined as follows:

- **CNN baseline:** an approach solely based on spatial information, without considering any elements related to time or graphs. The backbone DenseNet121 extracts the spatial information from the single image input, followed by flattening and passing the output through the multi-layer perceptron (MLP) regression head for lane coordinates regression. It forms the basic spatial capability of the model (Qin et al., 2020; Tabelini et al., 2021).
- **Temporal baseline:** this architectural design builds on the baseline CNN model by adding the Temporal Transformer module. Extracted features from a sequence of three frames (*t*, *t*−1, *t*−2) are pooled. But it does not have any kind of graph-based relational reasoning and is making predictions via a linear head (Bai et al., 2023). It isolates only the benefits of historical context
- **GCN baseline:** for examining the selection of graph reasoning in particular, the above architecture has been modified by substituting the dynamic graph attention layers with GCN layers that follow standard practice. (Kipf and Welling, 2017). The adjacency matrix is statically predefined as a 1D chain, connecting only immediately adjacent row-anchor neighbors without dynamic edge weighting.
- **Fixed-topology GAT:** while this architecture uses the same Graph Attention Network (GAT) layers (Veličković et al., 2017) as that of the proposed framework, it does not include Uncertainty Weighted DTGC module. The adjacency matrix is static and connected based on local neighbors, showing the need for learning dynamic edge weights in uncertain driving conditions.

The selection of these particular baseline architectures was done in order to demonstrate the need for each component of the proposed architecture. The CNN Baseline was chosen in order to determine the fundamental limitations of purely spatial feature extraction and to confirm the limitations of single frame detection in highly occluded areas. The Temporal Baseline was included to show that historical context helps, but temporal aggregation alone does not solve the problem due to the lack of geometrical reasoning. Next, the GCN Baseline was picked in order to show the limitation of classical message passing through graphs where fixed adjacency matrices do not take into account changing topologies of roads since all nodes are equal. Lastly, the Fixed-Topology GAT was included to examine that no matter how sophisticated an algorithm may be, the influence of noise cannot be filtered out if the architecture of the graph stays the same. Through carefully demonstrating the limitation of each architecture component, the need for including temporal, geometrical, and dynamic topological reasoning in one architecture becomes clear. The quantitative comparison results obtained using these baselines are presented in Table 8. To assess the localization performance of the proposed framework, the results are compared with the internal baselines using F1 and IoU as measures of lane-detection performance, while MAE, RMSE, and Average Localization Error (pixels) quantify coordinate-level localization accuracy. Published CULane methods such as LaneATT, CLRNet, and UFLDv2 provide useful reference points for the broader lane-detection literature; however, their reported scores are not directly reproduced in Table 8 because their original experimental protocols and output representations differ from the 25-row-anchor lane-localization formulation adopted in GraphLane. These methods are therefore considered as representative published approaches in the related-work context, while Table 8 is reserved for controlled comparisons under the same implementation and evaluation protocol.

The results presented in Table 8 show that the proposed framework achieves competitive overall localization performance among the evaluated models, while incurring higher computational cost. The proposed model achieves a competitive F1-score of 0.9363 and the highest IoU of 0.8803 among the evaluated models. Although the F1-score is slightly lower than that of the Fixed GAT model (0.9386), the proposed framework achieves substantially lower MAE (0.0241), RMSE (0.0464), and Average Localization Error (19.28 pixels). Compared with the CNN baseline, the proposed model improves the F1 score from 0.9308 to 0.9363 and reduces the pixel error from 29.73 to 19.28 pixels. These results indicate that the proposed framework provides more accurate coordinate localization while maintaining competitive lane-detection performance.

From a computational perspective, the proposed approach contains 3.81M parameters and operates at 88.64 FPS with a latency of 11.28 ms. Thus, it has a higher computational cost than the evaluated baseline models, particularly in terms of inference speed and latency. The CNN baseline provides the highest inference speed at 500.39 FPS and the lowest latency at 2.00 ms, reflecting its relatively simple architecture. However, its localization performance is lower than that of the proposed model, with an F1 score of 0.9308 and a pixel error of 29.73 pixels. The Temporal model achieves an F1 score of 0.9356 and an IoU of 0.8791, but its MAE and pixel error remain higher than those of the proposed framework, suggesting that temporal modeling alone does not provide the same coordinate-localization accuracy. The GCN baseline shows lower performance across the reported localization metrics, with an F1 score of 0.8764, an IoU of 0.7800, and a pixel error of 61.05 pixels. In comparison, the Fixed GAT model achieves an F1 score of 0.9386 and an IoU of 0.8897, which are close to the proposed framework. However, its MAE of 0.0389 and pixel error of 31.10 pixels are higher than those of the proposed model. Overall, the results suggest that the proposed framework provides improved coordinate-level localization relative to the evaluated baselines, although this improvement is accompanied by increased computational cost.

Qualitative comparison in Figures 9, 10 further supports the quantitative results mentioned earlier. In difficult nighttime conditions with heavy traffic in an urban environment, the basic model often provides results with fragmented lane structure, inaccurate lane geometry, and even false lane prediction. On the other hand, the proposed model creates smoother and more continuous lane structures on the actual road geometry. Overall, the quantitative results together with the qualitative comparisons indicate that the proposed framework provides more reliable lane localization than the considered baseline models. The integration of temporal feature aggregation, adaptive graph reasoning, and geometry-aware optimization enables the network to preserve lane continuity and produce geometrically consistent lane predictions across challenging driving scenarios. A limitation of the present study is the absence of a direct, protocol-matched experimental comparison with established CULane methods. Although representative published methods are discussed to provide contextual comparison, reproducing these methods under exactly the same implementation and evaluation conditions was beyond the scope of this study. Consequently, the reported results should be interpreted primarily as evidence of the effectiveness of the proposed architecture and its components under the evaluation formulation adopted in this work.

**Figure 9:**
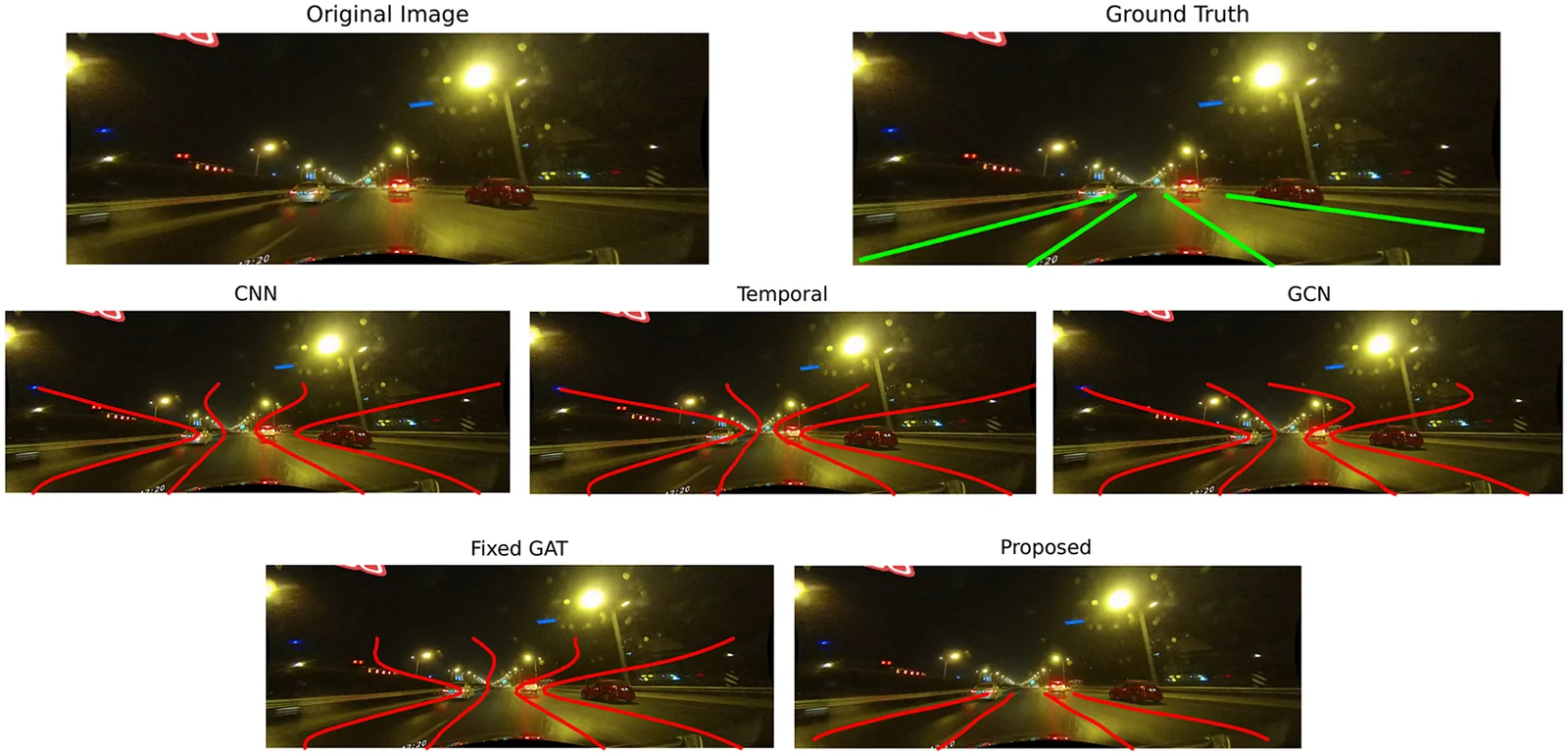
Qualitative comparison of lane localization performance under nighttime driving conditions. The proposed framework produces smoother and geometrically consistent lane predictions, whereas the baseline models exhibit fragmented lane structures, incorrect lane geometry, and false lane estimations under low-visibility conditions.

**Figure 10:**
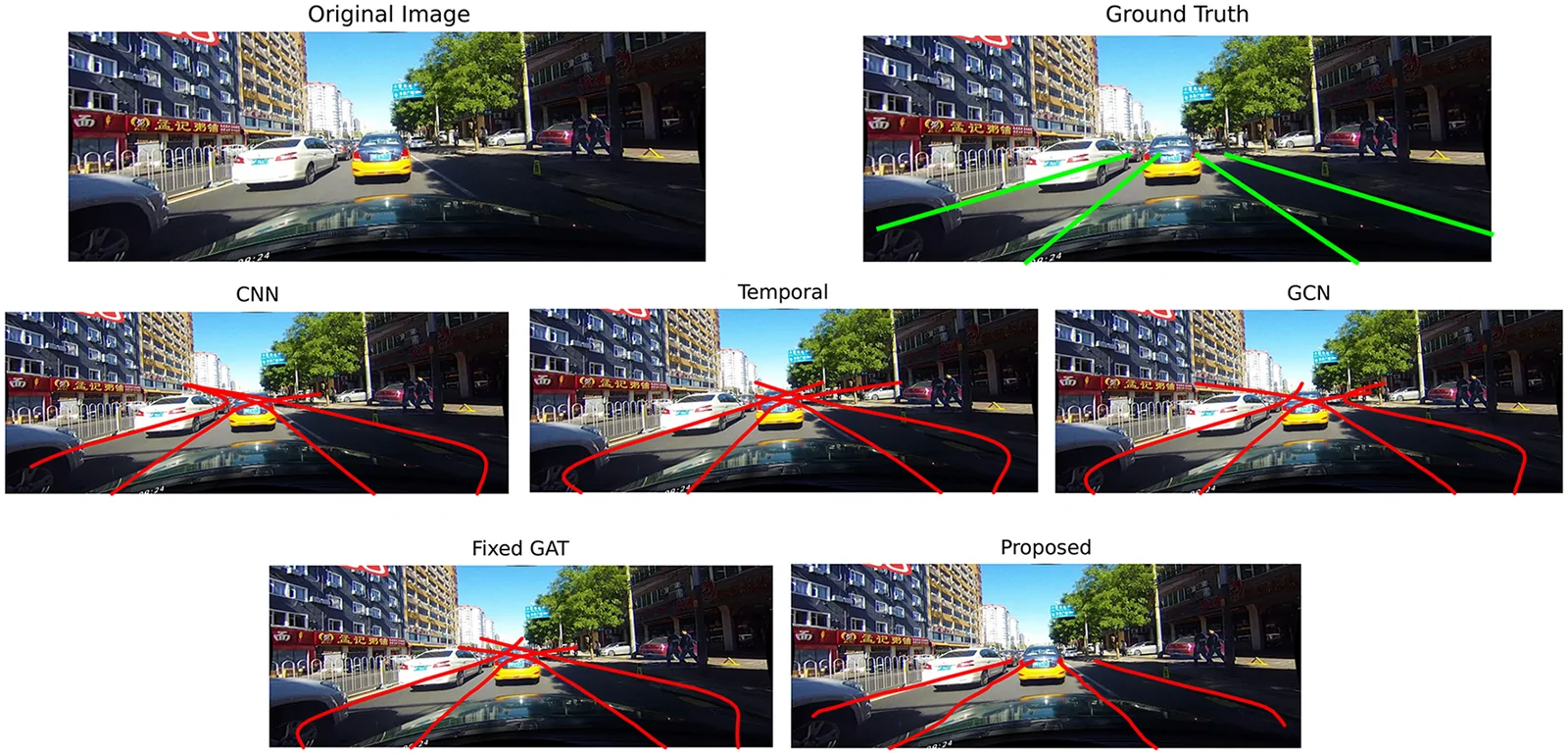
Qualitative comparison of lane localization performance in a complex urban traffic scene. The proposed framework maintains continuous lane geometry despite nearby vehicles and scene clutter, while the baseline models show degraded lane continuity and inaccurate lane localization.

### 6.8 Ablation study

Ablation experiments were conducted to evaluate the contribution of the proposed components to the overall performance of GraphLane. The ablation study follows an incremental design in which the proposed components are progressively introduced. The baseline configuration excludes Dynamic Temporal Graph Construction (DTGC), Confidence-Weighted Graph Construction (CWGC), Temporal Graph Consistency Loss (TGCL), and geometry-aware optimization. The subsequent configurations progressively introduce DTGC, CWGC, and TGCL, followed by the complete geometry-aware formulation. The component combinations evaluated in the ablation study are summarized in Table 9, where ✓ indicates that a component is enabled and × indicates that it is disabled.

**Table 9:** Component configurations used in the ablation study.

Configuration	DTGC	CWGC	TGCL	Geometry
Baseline	×	×	×	×
+DTGC	✓	×	×	×
+DTGC+CWGC	✓	✓	×	×
+DTGC+CWGC+TGCL	✓	✓	✓	×
Full Model without TGCL	✓	✓	×	✓
**Full framework**	✓	✓	✓	✓

Bold values indicate the proposed/full framework configuration or the selected default parameter setting.

The configurations therefore follow the primary progression Baseline → +DTGC → +DTGC + CWGC → +DTGC + CWGC + TGCL → full framework. In addition, the Full Model without TGCL configuration provides a separate controlled ablation in which TGCL is removed while DTGC, CWGC, and geometry-aware optimization are retained. This comparison allows the specific contribution of TGCL to be examined independently. All experiments were performed under identical training settings using the CULane dataset. All ablation configurations were trained using the training split, with model selection based on validation loss; the held-out CULane test split was used only for final evaluation. The performance results are summarized in Table 10.

**Table 10:** Ablation study of the proposed GraphLane framework.

Configuration	Precision	Recall	F1	Avg. localization error (px)
Baseline	0.98	0.8837	0.9319	27.70
+DTGC	0.9854	0.7556	0.8554	23.75
+DTGC + CWGC	0.9786	0.9067	0.9413	16.29
+DTGC + CWGC + TGCL	0.9737	0.8168	0.8884	28.75
Full model without TGCL	0.9895	0.7596	0.8595	17.24
Full framework	**0.9769**	**0.8990**	**0.9363**	**19.28**

Bold values indicate the proposed/full framework configuration or the selected default parameter setting.

The ablation results in Table 10 demonstrate that the proposed components contribute differently to lane-existence detection and coordinate-level localization. Rather than exhibiting a strictly monotonic improvement after each component is introduced, the results reveal complementary effects and interactions among dynamic graph construction, uncertainty-based graph weighting, temporal consistency, and geometry-aware optimization.

The baseline configuration achieved a precision of 0.9800, a recall of 0.8837, an F1-score of 0.9319, and an Average Localization Error of 27.70 pixels. Although the baseline provides a relatively strong lane-existence F1-score, its higher localization error indicates that accurate lane existence prediction does not necessarily imply precise coordinate localization. This provides the reference point for evaluating the contribution of the subsequent components.

Adding Dynamic Temporal Graph Construction (DTGC) increased precision from 0.9800 to 0.9854 and reduced the Average Localization Error from 27.70 to 23.75 pixels, corresponding to an approximately 14.3% reduction in localization error. However, recall decreased from 0.8837 to 0.7556, resulting in a lower F1-score of 0.8554. This indicates that DTGC primarily improved coordinate localization in this configuration, while its sparse dynamic connectivity also made the model more selective in its lane-existence predictions. Thus, dynamic graph construction alone does not provide the best balance between localization accuracy and lane-existence detection.

The addition of Confidence-Weighted Graph Construction (CWGC) produced a substantial improvement over DTGC alone. Precision decreased slightly from 0.9854 to 0.9786, but recall increased substantially from 0.7556 to 0.9067, raising the F1-score from 0.8554 to 0.9413. At the same time, the Average Localization Error decreased from 23.75 to 16.29 pixels, representing an approximately 31.4% reduction relative to the DTGC configuration. This result indicates that uncertainty-derived confidence weighting helps suppress the influence of unreliable node interactions while preserving useful relational information, leading to improvements in both lane-existence detection and coordinate localization. Among the configurations without geometry-aware optimization, the DTGC+CWGC configuration therefore provides the strongest overall balance.

The effect of Temporal Graph Consistency Loss (TGCL) is more configuration dependent. When TGCL was added to the DTGC + CWGC configuration without geometry-aware optimization, the F1-score decreased from 0.9413 to 0.8884 and the Average Localization Error increased from 16.29 to 28.75 pixels. This indicates that the incremental addition of TGCL did not improve performance in this particular configuration. However, this result should not be interpreted as evidence that TGCL is ineffective in the complete framework, because the effect of TGCL depends on its interaction with geometry-aware optimization.

The specific contribution of geometry-aware optimization can be examined by comparing the “+DTGC + CWGC + TGCL” configuration with the Full Framework, since both configurations contain DTGC, CWGC, and TGCL and differ only in the geometry-aware optimization component. Introducing geometry-aware optimization increased recall from 0.8168 to 0.8990 and F1-score from 0.8884 to 0.9363, while reducing the Average Localization Error from 28.75 to 19.28 pixels. This corresponds to an approximately 32.9% reduction in localization error. The result demonstrates that geometry-aware constraints provide an important complement to graph and temporal reasoning by improving the structural consistency and localization of the predicted lanes.

The contribution of TGCL can be further examined independently by comparing the Full Model without TGCL with the Full Framework. In this comparison, DTGC, CWGC, and geometry-aware optimization are kept unchanged, while TGCL is the only component that differs. Adding TGCL increases recall from 0.7596 to 0.8990 and F1-score from 0.8595 to 0.9363. Although precision decreases from 0.9895 to 0.9769 and the Average Localization Error increases from 17.24 to 19.28 pixels, the substantial improvement in recall and F1-score indicates that TGCL improves the temporal robustness of lane-existence predictions in the complete geometry-aware formulation. This controlled comparison therefore provides stronger evidence for the contribution of TGCL than the incremental TGCL comparison alone.

Overall, the ablation results indicate that the proposed components serve different and complementary purposes. DTGC improves relational localization but can make lane-existence predictions more selective when used alone, while CWGC substantially improves the balance between detection and localization by incorporating uncertainty into graph interactions. TGCL has a configuration- dependent effect, with its strongest contribution becoming evident when combined with the complete geometry-aware formulation. Geometry-aware optimization substantially improves the final balance between lane-existence detection and localization when applied to the graph-temporal configuration. The Full Framework consequently achieves a precision of 0.9769, recall of 0.8990, F1-score of 0.9363, and Average Localization Error of 19.28 pixels. These results support the interpretation that the effectiveness of GraphLane arises from the interaction of its components rather than from any single component acting independently.

### 6.9 Sensitivity analysis

To further investigate the robustness of GraphLane, sensitivity analyses were conducted by varying the principal hyperparameters associated with two key components of GraphLane: geometry-aware optimization and Dynamic Temporal Graph Construction (DTGC). All sensitivity configurations were trained using the complete CULane training split and evaluated exclusively on the complete validation split containing 9,855 images. The test split was not used for sensitivity analysis and was reserved for the final evaluation.

The influence of the geometry-aware optimization was examined by varying the smoothness (λ_*s*_) and topology (λ_*t*_) loss weights while keeping all remaining loss terms unchanged. As shown in Table 11, the default configuration achieved the highest F1-score of 0.9330 and the lowest Average Localization Error of 19.90 pixels. Reducing or increasing the smoothness and topology weights resulted in lower F1-scores and higher localization errors relative to the default configuration. These results indicate that the selected weighting provides a favorable balance between coordinate regression and geometric regularization under the validation-based sensitivity evaluation. These results indicate that the selected multi-objective loss configuration provides an effective balance between coordinate regression and geometric regularization, thereby improving both detection performance and localization accuracy.

**Table 11:** Sensitivity analysis of the proposed GraphLane framework.

Configuration	F1-score	Average localization error (pixels)
Geometry-aware optimization		
Default (λ_*s*_ = 0.30, λ_*t*_ = 0.15)	**0.9330**	**19.90**
λ_*s*_ = 0.20	0.9003	24.86
λ_*s*_ = 0.40	0.9054	27.31
λ_*t*_ = 0.10	0.9189	25.82
λ_*t*_ = 0.20	0.9171	22.94
Dynamic Temporal Graph Construction		
*k* = 4	0.9238	23.73
*k* = 6	0.8970	25.78
*k* = 8 (Default)	**0.9330**	**19.90**
*k* = 10	0.9327	23.85

Bold values indicate the proposed/full framework configuration or the selected default parameter setting.

The sensitivity of the DTGC module was further investigated by varying the Top-*k* sparsification parameter, which determines the number of graph connections preserved for each node before graph attention reasoning. As shown in Table 11, the tested values produced relatively stable F1-scores, although the localization error varied across different sparsity levels. The most sparse configuration (*k* = 4) achieved an F1-score of 0.9238 with an Average Localization Error of 23.73 pixels, while *k* = 6 produced the lowest F1-score of 0.8970. The default setting (*k* = 8) achieved the highest F1-score of 0.9330 and the lowest localization error of 19.90 pixels among the tested configurations. Increasing the neighborhood to *k* = 10 resulted in a similar F1-score of 0.9327 but a higher localization error of 23.85 pixels. These results support the selection of *k* = 8 as a balanced configuration for graph sparsity and localization performance.

### 6.10 Discussion

The experimental results consistently demonstrate that accurate lane localization requires more than conventional pixel-wise feature extraction or static graph reasoning. The experimental results indicate that GraphLane provides a favorable balance between lane-existence detection and coordinate localization under the proposed evaluation protocol, particularly in terms of coordinate-level localization. Unlike other lane detection systems that mainly focus on the accuracy of classification, the proposed system takes into account lane structure and temporal consistency which greatly reduces localization errors.

In terms of quantitative evaluation, the proposed approach provides consistent lane-existence and coordinate localization performance under the row-anchor-based evaluation adopted in this study. On the study evaluation split, GraphLane achieved a lane-existence F1-score of 0.9363 and an Average Localization Error of 19.28 pixels. On the complete official CULane test split, the corresponding values were 0.9013 and 42.34 pixels, respectively. The difference reflects the use of the larger complete test split and the greater diversity of its scene conditions rather than a change in model configuration. These results provide evidence of the behavior of the proposed architecture under the adopted evaluation formulation, but they should not be interpreted as official CULane benchmark scores. The high F1-score demonstrates that combining temporal context with graph reasoning improves lane continuity, reducing fragmented detections that commonly occur in occluded scenes. The low localization error indicates that the geometry-aware optimization effectively constrains coordinate regression, producing smoother and more structurally consistent lane predictions.

The qualitative results further demonstrate how GraphLane ensures the persistence of the lane geometry in various difficult scenarios,such as occlusions, shadows, heavy traffic, nighttime environment, and drastic illumination changes. As opposed to spatial-only methods, which frequently result in discontinuity of the lane predictions in partially visible environments, the suggested approach promotes more persistent lane predictions by integrating temporal feature aggregation and graph-relational reasoning. In addition, the confidence estimation technique is helpful in eliminating lane predictions in those cases where there are no lane markings present.

The dynamic graph analysis further examines whether the learned graph topology varies across different driving conditions. Although the Top-*k* value is fixed at 8 and therefore constrains the graph sparsity, the identities of the selected edges are not fixed across input samples. As shown in Table 12, using the official CULane test-condition lists, the mean edge-change rate ranges from 31.28% for Crowd scenes to 50.84% for Arrow scenes, with corresponding mean Jaccard similarities ranging from 0.4916 to 0.6872. The largest edge variation is observed for Arrow (50.84%) and Curve (43.96%) conditions, while substantial graph variation is also observed for Normal (37.45%), Noline (35.87%), and Shadow (34.65%) scenes. These results indicate that the selected graph connections vary across input samples and driving conditions despite the fixed Top-*k* sparsification, providing empirical evidence of sample- and condition-dependent graph construction in the proposed DTGC module.

**Table 12:** Condition-wise graph adaptation analysis on the CULane test set.

Condition	Samples	Mean Jaccard	Edge change (%)
Normal	9,621	0.6255	37.45
Crowd	8,113	0.6872	31.28
Hlight	486	0.6864	31.36
Shadow	930	0.6535	34.65
Noline	4,067	0.6413	35.87
Arrow	890	0.4916	50.84
Curve	422	0.5604	43.96
Cross	3,122	0.6767	32.33
Night	7,029	0.6809	31.91

The results of the ablation study show how the contribution of each component to the final performance varies. DTGC makes the largest contribution at the first step of localization error reduction due to the ability of the adaptive information exchange between lane keypoints. Confidence-weighted graph construction suppresses unreliable node interactions, improving information propagation during graph reasoning and leading to more accurate coordinate estimation. Despite providing little contribution to localization accuracy improvement, TGCL creates temporal consistency between consecutive graphs. The controlled comparison further shows that geometry-aware optimization substantially improves the balance between lane-existence detection and localization in the graph-temporal configuration.

From a practical perspective, the computational analysis shows that the proposed framework achieves 88.64 FPS with a latency of 11.28 ms per forward pass under the specified Tesla P100 evaluation setup. These measurements indicate real-time-capable inference under the tested GPU configuration; however, they do not by themselves establish suitability for deployment on in-vehicle hardware. Evaluation across automotive-grade and embedded computing platforms is required to assess deployment feasibility under practical vehicle operating conditions.

#### 6.10.1 Limitations

Despite the localization performance of the proposed GraphLane, the system is still based on fixed row-anchors and requires supervised lane annotations. Additionally, the presented model was evaluated on one dataset, CULane, and its computational performance was measured on a Tesla P100 GPU. Therefore, the reported inference speed should not be interpreted as evidence of deployment performance on automotive or embedded hardware. Future research will focus on inter-dataset generalization, evaluation on diverse hardware platforms, lightweight deployment, and self-supervised learning of temporal graphs.

In summary, all the experiment results provide strong support to the main hypothesis of this research work, which is that “lane localization through accurate lane detection can be improved by incorporating temporal context, dynamic graph structure, confidence-weighted graph reasoning and geometry constraint models.” Contrary to viewing lane detection merely as a classification problem, GraphLane considers lane detection as a structured spatio-temporal localization problem and therefore can achieve lane localization more accurately in varied driving scenarios.

## 7 Conclusion

This paper presented a unified spatio-temporal graph learning framework for robust lane localization by integrating spatial feature extraction, temporal context aggregation, confidence-weighted dynamic graph construction, graph attention reasoning, and geometry-aware optimization within a single end-to-end architecture. The proposed model was designed to improve lane coordinate localization while maintaining reliable lane detection across challenging driving environments. The experiments conducted on the CULane dataset achieved an Average Localization Error of 19.28 pixels and a lane-existence F1-score of 0.9363 under the row-anchor-based evaluation defined in this study. The proposed framework produces a consistent and smooth output in case of shadows, occlusions, traffic, and night scenes. The analysis of dynamic graphs demonstrates the sparse and temporally consistent nature of learned graphs while adjusting according to the changes in the scene. Ablation experiments show that the proposed components contribute differently to lane-existence detection and coordinate localization, with their effects depending on the overall configuration. Moreover, the computational analysis shows that the proposed framework contains 3.81 million trainable parameters and achieves 88.64 FPS with a latency of 11.28 ms per forward pass under the tested Tesla P100 GPU configuration. These results demonstrate real-time-capable inference under the reported experimental setup, while broader evaluation on automotive and embedded hardware remains necessary to establish deployment feasibility. Future work will focus on extending the framework to more diverse driving datasets and adverse weather conditions, investigating cross-dataset generalization, and integrating the proposed lane localization framework with downstream autonomous driving tasks such as trajectory planning and vehicle control.

## Data Availability

The dataset used in this study is publicly available. The experiments were conducted using the CULane dataset, which is available from the official CULane benchmark. For ease of experimentation, the dataset was accessed through the Kaggle mirror: https://www.kaggle.com/datasets/manideep1108/culane.
